# Investigating the impact of multidimensional sleep traits on cardiovascular diseases and the mediating role of depression

**DOI:** 10.1136/openhrt-2024-002866

**Published:** 2025-03-13

**Authors:** Hao Zhao, Xiaojie Wang, Lan Guo, Xiuwen Li, Kayla M Teopiz, Roger S McIntyre, Wanxin Wang, Ciyong Lu

**Affiliations:** 1 Department of Medical Statistics and Epidemiology, School of Public Health, Sun Yat-sen University, Guangzhou, China; 2 Guangdong Provincial Key Laboratory of Food, Nutrition and Health, Sun Yat-Sen University, Guangzhou, China; 3 Department of Neurology, Shenzhen Shekou People’s Hospital, Shenzhen, China; 4 Mood Disorders Psychopharmacology Unit, University Health Network, Toronto, Ontario, Canada; 5 Department of Psychiatry, University of Toronto, Toronto, Ontario, Canada

**Keywords:** PUBLIC HEALTH, Genome-Wide Association Study, EPIDEMIOLOGY, RISK FACTORS, Coronary Artery Disease

## Abstract

**Background:**

Observational studies have reported that sleep is associated with the risk of major depressive disorder (MDD) and cardiovascular diseases (CVDs). However, the causal relationships among various sleep traits remain contentious, and whether MDD mediates the impact of specific sleep traits on CVDs is unclear.

**Methods:**

We performed two-sample Mendelian randomisation analyses to explore whether insomnia, sleep time, daytime napping, daytime sleepiness, chronotype, snoring or obstructive sleep apnoea were causally associated with the risk of five CVDs, including coronary artery disease (CAD), myocardial infarction (MI), heart failure (HF), atrial fibrillation and stroke. Mediation analyses were performed to assess the proportion mediated by MDD.

**Results:**

Genetically predicted insomnia, short sleep, daytime napping and daytime sleepiness increased the risk of CVDs, with the OR ranging from 1.24 (95% CI 1.06 to 1.45) for insomnia on stroke to 1.55 (95% CI 1.28 to 1.89) for insomnia on MI. In contrast to short sleep, genetically predicted sleep duration decreased the risk of CAD (OR 0.88 (95% CI 0.80 to 0.97)), MI (OR 0.89 (95% CI 0.80 to 0.99)) and HF (OR 0.90 (95% CI 0.83 to 0.98)). However, we found no significant associations of long sleep, chronotype, snoring and obstructive sleep apnoea with increased risk for any CVD subtype. Additionally, the effect of insomnia was partially mediated by MDD for the risk of CAD (proportion mediated: 8.81% (95% CI 1.20% to 16.43%)), MI (9.17% (95% CI 1.71% to 16.63%)) and HF (14.46% (95% CI 3.48% to 25.45%)). Similarly, the effect of short sleep was partially mediated by MDD for the risk of CAD (8.92% (95% CI 0.87% to 16.97%)), MI (11.43% (95% CI 0.28% to 22.57%)) and HF (12.65% (95% CI 1.35% to 23.96%)). MDD also partially mediated the causal effects of insomnia on stroke, sleep duration on CAD, MI and HF, daytime napping on HF and daytime sleepiness on CAD.

**Conclusions:**

Our study provides evidence that genetically predicted insomnia, short sleep, frequent daytime napping and sleepiness are associated with a higher risk of certain CVD subtypes, partly mediated by MDD.

**WHAT IS ALREADY KNOWN ON THIS TOPIC**

**WHAT THIS STUDY ADDS**

- This study provides robust genetic evidence from MR analyses, showing that insomnia, short sleep, frequent daytime napping and sleepiness are causally linked to an increased risk of various CVDs. It also demonstrates that MDD partially mediates the effects of insomnia and short sleep on coronary artery disease, myocardial infarction and heart failure, with mediation proportions ranging from 8.81% to 14.46%.

**HOW THIS STUDY MIGHT AFFECT RESEARCH, PRACTICE OR POLICY**

- The findings underscore the importance of considering sleep traits and MDD in the prevention and screening of CVDs. Health policies should emphasise early detection and treatment of MDD in individuals with poor sleep patterns to mitigate CVD risks.

## Introduction

 Cardiovascular diseases (CVDs) are the leading cause of death and a major contributor to the global burden of disease. Currently, over 600 million individuals are affected by CVDs, with this number steadily increasing.^1^ With the global population ageing rapidly, CVDs are expected to lead to even greater losses in healthy life years and further exacerbate the associated socioeconomic burden. In addition to some established risk factors (eg, hypertension, unhealthy diet, hyperlipidaemia, air pollution, smoking, hyperglycaemia and obesity), many studies have also shown that sleep plays an important role in CVDs.^2^,^6^ Sleep, as a modifiable lifestyle factor, is closely linked to circadian rhythms. Multidimensional sleep traits include insomnia, sleep duration, daytime napping, daytime sleepiness, chronotype (also known as circadian preference), snoring and obstructive sleep apnoea. However, findings from observational studies on sleep traits and CVDs have been inconsistent. For example, some studies suggest that napping increases cardiovascular risk,^7 8^ while others indicate that napping may protect against CVD.^9^ These discrepancies are likely due to uncertain temporal relationships, insufficient sample sizes, short follow-up periods or potential confounding factors.

Mendelian randomisation (MR), which uses genetic variation from single-nucleotide polymorphisms (SNPs) of the exposure as instrumental variables (IVs) to minimise measurement errors, confounding and reverse causality, can provide a reliable estimation of the causal association between exposure and outcome under specific assumptions (relevance, independence and exclusion restriction). Current research on sleep and CVDs focuses on insomnia and sleep duration, while other sleep phenotypes remain understudied. A previous study showed that being a morning person may be a potential ‘risk factor’ for cardiometabolic diseases based on MR analyses.^10^ However, they failed to exclude SNPs associated with confounding factors, which could lead to horizontal pleiotropic bias resulting in inaccurate findings. Therefore, there is still a lack of comprehensive research on the causal associations between multidimensional sleep traits and CVDs, including coronary artery disease (CAD), myocardial infarction (MI), heart failure (HF), atrial fibrillation (AF) and stroke, at the genetic level.

Several well-recognised risk factors for CVDs appear to be mediators for sleep traits. For example, Liu *et al*^11^ assessed 17 cardiometabolic risk factors but did not consider major depressive disorder (MDD) and reported that body mass index and triglycerides accounted for 14.97% and 11% of the causal effect of insomnia on CAD, respectively. However, this means that a significant mediated proportion of the association between sleep traits and CVDs remains unexplained. Current evidence suggests that there is an association between sleep and MDD, as well as between MDD and CVDs. Although the associations among these three may be intricate and perhaps interdependent, sleep problems due to MDD and psychological problems due to CVDs have been supported by multiple studies.^12 13^ However, whether sleep problems can lead to MDD and further promote CVDs has not been adequately studied. Hall *et al*^14^ presented a conceptual model of the possible influence of MDD on the relationship between sleep and CVDs but did not conduct quantitative analyses. Moreover, conventional observational studies exploring sleep, MDD and CVDs have so far been inconclusive, as observational evidence may be subject to confounding and reverse causality, especially in mediation studies, which may be subject to measurement error and collider bias. Therefore, it is unclear whether MDD could explain some of the mechanisms underlying the effect of sleep traits on CVDs. Fortunately, the mediation analysis of the two-step MR can yield unbiased causal estimates at the genetic level.

In this study, we tested two hypotheses by univariable, multivariable and two-step two-sample MR analyses. First, we aimed to evaluate whether there is a potential causal association between multidimensional sleep traits and MDD and CVDs. Second, we aimed to evaluate whether MDD mediates the effect of sleep traits on CVDs.

## Methods

### Study design

**Figure 1:**
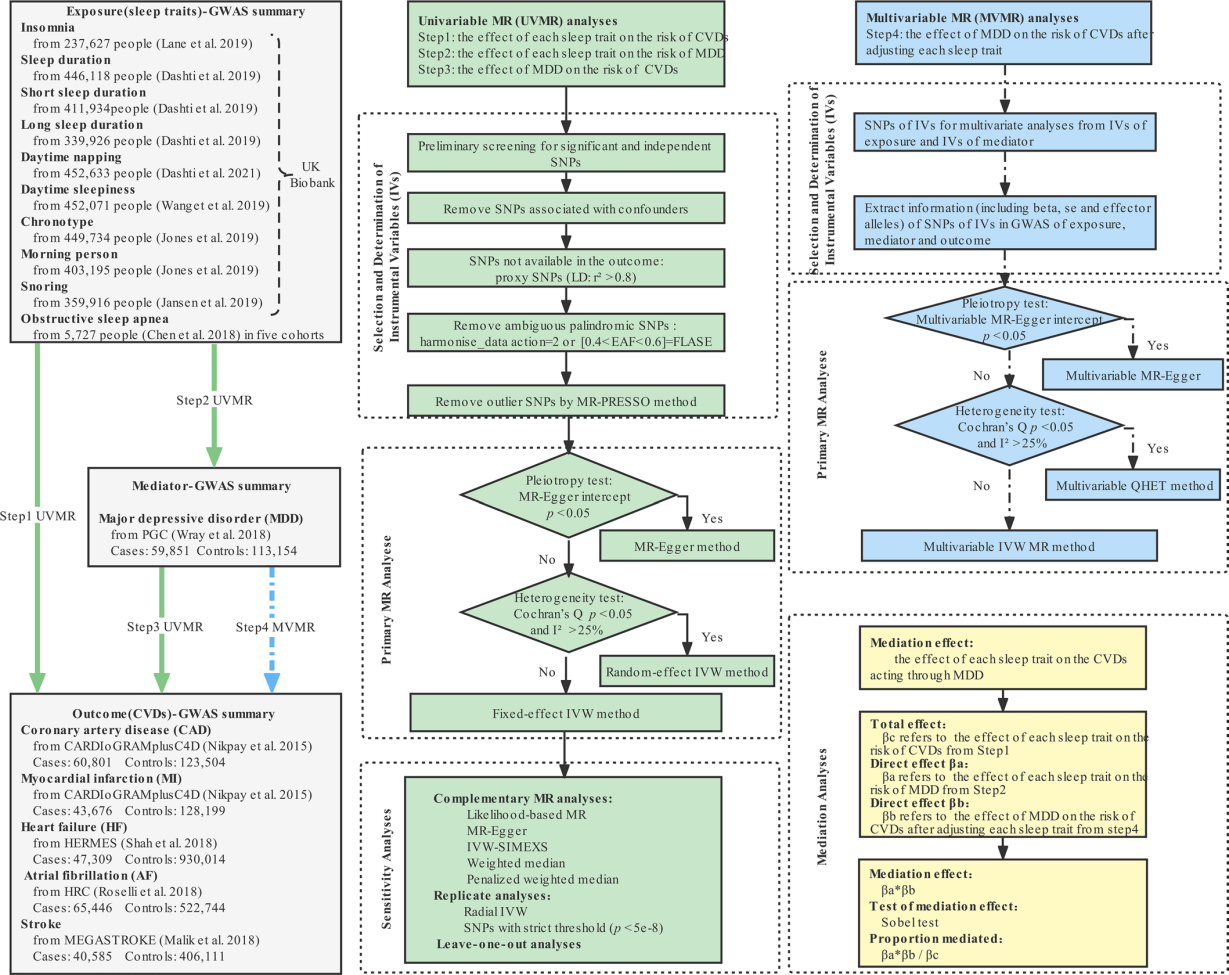
The flow chart of MR analyses in this study. CARDIoGRAMplusC4D, Coronary Artery Disease Genome-wide Replication and Meta-analysis (CARDIoGRAM) plus The Coronary Artery Disease (C4D) Genetics; CVDs, cardiovascular diseases; EAF, effect allele frequency; HERMES, Heart Failure Molecular Epidemiology for Therapeutic Targets; HRC, Haplotype Reference Consortium; IVW, inverse-variance weighted; LD, linkage disequilibrium; PGC, the psychiatric Genomics Consortium; SNP, single-nucleotide polymorphism.

We performed univariable, multivariable and two-step two-sample MR mediation analyses to investigate whether genetically predicted sleep traits were causally associated with the risk of five CVDs, including CAD, MI, HF, AF and stroke, and to assess the proportion mediated by MDD in the above associations. The flow chart of MR analyses is shown in figure 1. This study is conducted and reported according to the Strengthening the Reporting of Observational Studies in Epidemiology Using MR checklist.

### GWAS data for sleep

We examined multidimensional sleep traits as exposures, closely related to circadian rhythms. These traits included insomnia,^15^ sleep duration,^16^ short sleep (≤6 hours),^16^ long sleep (≥9 hours),^16^ daytime napping,^17^ daytime sleepiness,^18^ chronotype,^19^ morning person,^19^ snoring^20^ and obstructive sleep apnoea (quantified using the apnoea-hypopnoea index).^21^ The GWAS data of these sleep traits were from publicly available summary statistics, mainly based on the UK Biobank.^22^ The data sources and information for GWAS are described in detail in online supplemental eMethod 1.

### GWAS data for depression

For MDD, we used the available summary statistics of MDD-GWAS of European ancestry (59 851 MDD cases and 113 154 controls) recently released by the psychiatric Genomics Consortium (PGC), including PGC29 and the five additional cohorts.^23^ Based on the international diagnostic criteria (Diagnostic and Statistical Manual of Mental Disorders, Third Edition [DSM-III], Fourth Edition [DSM-IV], International Classification of Diseases, Ninth Revision [ICD-9], or Tenth Revision [ICD-10]), the diagnosis of MDD was determined through a structured interview, clinician-administered checklists or medical record reviews. Exclusion criteria included cases with lifetime bipolar disorder or schizophrenia and controls with a history of MDD.

### GWAS data for CVDs

We used publicly available summary statistics of GWAS for CAD and MI across 48 studies from the Coronary Artery Disease Genome-wide Replication and Meta-analysis plus The Coronary Artery Disease Genetics (CARDIoGRAMplusC4D) consortium (77% European ancestry, CAD: 60 801 cases and 123 504 controls; MI: 43 676 cases and 128 199 controls),^24^ HF across 29 studies from the Heart Failure Molecular Epidemiology for Therapeutic Targets Consortium (all European ancestry, 47 309 cases and 930 014 controls),^25^ AF from Haplotype Reference Consortium (HRC) (91% European ancestry, 65 446 cases and 522 744 controls)^26^ and stroke from the MEGASTROKE consortium (all European ancestry, 40 585 cases and 406 111 controls).^27^

### Statistical analysis

The two-sample MR analysis is an approach that uses a set of SNPs as IVs to obtain estimates for the causal effect of exposure on the outcome, where the IVs need to satisfy three assumptions: the relevance assumption, the independence assumption and the exclusion restriction assumption (online supplemental figure 1). Details on the selection of genetic instruments are provided in online supplemental eMethod 2. The primary method for univariate MR analyses was determined based on pleiotropy and heterogeneity. First, the MR-Egger intercept test was performed to test whether there was the presence of potential pleiotropy.^28^ If there was significant horizontal pleiotropy, the MR-Egger regression was used; otherwise, the inverse-variance weighted (IVW) meta-analysis was used, which assumes that either all the instruments are valid or any horizontal pleiotropy is balanced. Then, heterogeneity was assessed using Cochran’s Q-statistics test.^29^ If there was significant heterogeneity (I^2^>25% and p<0.05 were considered statistically significant), the random-effect IVW model was used; otherwise, the fixed-effect IVW model was used. For IVs used in multivariate MR analyses, first, SNPs of IVs were derived from the combination of SNPs of IVs for each exposure and mediator in univariable MR analyses. Then, we extracted information (including beta, SE and effect alleles) of these SNPs of IVs from GWASs of exposure, mediator and outcome and harmonised effect allele. For multivariable MR analyses, the multivariable MR-Egger intercept test was performed to test for potential pleiotropy. If there was significant pleiotropy, the multivariable MR-Egger method was used for MR analyses. Then, when there was no significant pleiotropy, I^2^ and Cochran’s Q were used to further assess whether there was heterogeneity. If there was significant heterogeneity, the multivariable QHET method was used; otherwise, the multivariable IVW method was used for MR analyses. Sensitivity analyses including complementary MR methods and leave-one-out analysis are shown in online supplemental eMethod 2.

To estimate the mediation effect (ie, indirect effect), we estimated the effect of each exposure (ie, sleep traits) on the mediator (ie, MDD) individually using univariable MR analyses, then we multiplied this with the effect of the mediator (ie, MDD) on each outcome (ie, CVDs) after adjusting for each exposure (ie, sleep traits) using multivariable MR analyses.^30^ Finally, we divided the mediation effect by the total effect to estimate the proportion mediated. The steps and related formulas for mediation analyses of MR study are described in detail in online supplemental eMethod 3.

As many MR analyses with multiexposures and multioutcomes did, to account for multiple testing and to preserve the type I error of the global null hypothesis of all tested associations being, in fact, null, we used the Benjamini-Hochberg method to control the false discovery rate (FDR), with q<0.05 for FDR as significant evidence of associations, and between q>0.05 for FDR and uncorrected p<0.05 as suggestive evidence of associations. All statistical analyses were performed using R V.4.1.0.

## Results

### Selection of IVs for each MR analysis

The characteristics and sample sizes of the GWAS data sources are shown in table 1. Final IVs for each specific exposure in the MR analyses of different outcomes were identified after excluding candidate SNPs associated with confounders, missing in the outcome, ambiguous palindrome or outliers (online supplemental tables 1–3). The statistical power in the MR study suggested that for most analyses we had adequate statistical power to identify even modest causality (online supplemental table 4).

**Table 1:** GWAS data sources and information included in the current study

Trait	Phenotype	GWAS data source	Sample size	Definition (units)
Exposures	Insomnia	UK Biobank;Lane *et al*, 2019^15^	237 627	Binary variable of usually vs never/rarely (log-odds)
	Sleep duration	UK Biobank;Dashti *et al*, 2019^16^	446 118	Continuous variable(hours per day)
	Short sleep duration	UK Biobank;Dashti *et al*, 2019^16^	411 934	Binary variable of ≤6 hours per night vs 7–8 hours per night (log-odds)
	Long sleep duration	UK Biobank;Dashti *et al*, 2019^16^	339 926	Binary variable of ≥9 hours per night vs 7–8 hours per night (log-odds)
	Daytime napping	UK Biobank;Dashti *et al*, 2021^17^	452 633	Ordered categorical variable of never/rarely, sometimes, usually (more napping)
	Daytime sleepiness	UK Biobank;Wang et *al*, 2019^18^	452 071	Ordered categorical variable of never, sometimes, often, all the time (more sleepiness)
	Chronotype	UK Biobank;Jones *et al*, 2019^19^	449 734	Ordered categorical variable of definitely a morning person, more a morning than evening person, do not know, more an evening than morning person and definitely an evening person (more morningness)
	Morning person	UK Biobank;Jones *et al*, 2019^19^	403 195	Binary variable of the morning preference vs the evening preference (log-odds)
	Snoring	UK Biobank;Jansen *et al*, 2019^20^	359 916	Binary variable of yes vs no(log-odds)
	Apnoea–hypopnoea index	Five cohorts;Chen *et al*, 2018^21^	5727	Continuous variable(events per hour)
Mediator	MDD	PGC;Wray *et al*, 2018^23^	173 005	Binary variable of yes vs no(log-odds)
Outcomes	CAD	CARDIoGRAMplusC4D;Nikpay *et al*, 2015^24^	184 305	Binary variable of yes vs no(log-odds)
	MI	CARDIoGRAMplusC4D;Nikpay *et al*, 2015^24^	171 875	Binary variable of yes vs no(log-odds)
	HF	HERMES;Shah *et al*, 2020^25^	977 323	Binary variable of yes vs no(log-odds)
	AF	HRC;Roselli *et al*, 2018^26^	588 190	Binary variable of yes vs no(log-odds)
	Stroke	MEGASTROKE;Malik *et al*, 2018^27^	446 696	Binary variable of yes vs no(log-odds)

AF, atrial fibrillation; CAD, coronary artery disease; CARDIoGRAMplusC4D, Coronary Artery Disease Genome-wide Replication and Meta-analysis (CARDIoGRAM) plus The Coronary Artery Disease (C4D) Genetics; HERMES, Heart Failure Molecular Epidemiology for Therapeutic Targets; HF, heart failure; HRC, Haplotype Reference ConsortiumMDD, major depressive disorder; MI, myocardial infarction; PGC, the psychiatric Genomics Consortium

### The effect of each sleep trait on the risk of CVDs

**Figure 2:**
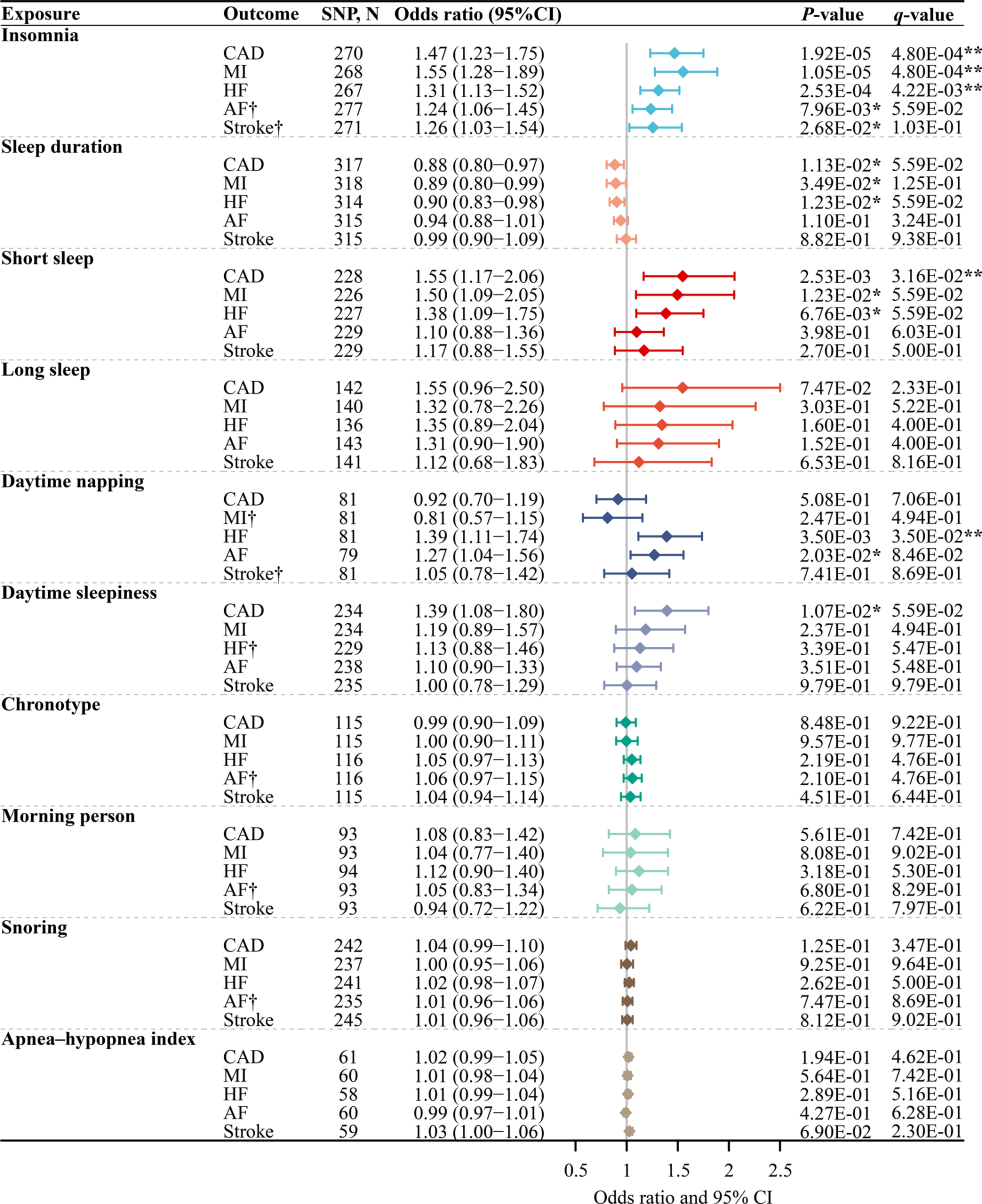
The main MR analysis results of the causal effects of sleep traits on CVDs. The OR represented the effect of genetically predicted per unit increase in each sleep trait. **Significant evidence (q-value<0.05) after correction for multiple testing estimated by the FDR method. *Suggestive evidence (uncorrected p<0.05 and q-value≥0.05). †Because there was significant heterogeneity, the random-effect IVW model was used, while for others, because there was no significant heterogeneity, the fixed-effect IVW model was used. AF, atrial fibrillation; CAD, coronary artery disease; CVD, cardiovascular disease; HF, heart failure; IVW, inverse-variance weighted; MI, myocardial infarction; SNP, single-nucleotide polymorphism.

The MR-Egger intercept test confirmed the absence of significant horizontal pleiotropy (p>0.05) (online supplemental table 5). Figure 2 shows that among 50 pairs of genetically predicted 10 sleep traits and 5 CVDs, 5 pairs were significant positive correlation, 6 pairs were suggestive positive correlation and 3 pairs were suggestive negative correlation. Genetically predicted per log odds increase in insomnia was associated with significantly increased risks of CAD (OR 1.47 (95% CI 1.23 to 1.75)), MI (OR 1.55 (95% CI 1.28 to 1.89)) and HF (OR 1.31 (95% CI 1.13 to 1.52)) and suggestively increased risks of AF (OR 1.24 (95% CI 1.06 to 1.45)) and stroke (OR 1.26 (95% CI 1.03 to 1.54)). Genetically predicted per hour/day increase in sleep duration was associated with suggestively decreased risks of CAD (OR 0.88 (95% CI 0.80 to 0.97)), MI (OR 0.89 (95% CI 0.80 to 0.99)) and HF (OR 0.90 (95% CI 0.83 to 0.98)). Likewise, genetically predicted per log odds increase in short sleep was associated with significantly increased risks of CAD (OR 1.55 (95% CI 1.17 to 2.06)) and suggestively increased risk of MI (OR 1.50 (95% CI 1.09 to 2.05)) and HF (OR 1.38 (95% CI 1.09 to 1.75)). Genetically predicted per category increase in daytime napping frequency was associated with a significantly increased risk of HF (OR 1.39 (95% CI 1.11 to 1.74)) and suggestively increased risk of AF (OR 1.27 (95% CI 1.04 to 1.56)). Similarly, genetically predicted per category in daytime sleepiness frequency was associated with a suggestively increased risk of CAD (OR 1.39 (95% CI 1.08 to 1.80)).

### The effect of each sleep trait on the risk of MDD

**Figure 3:**
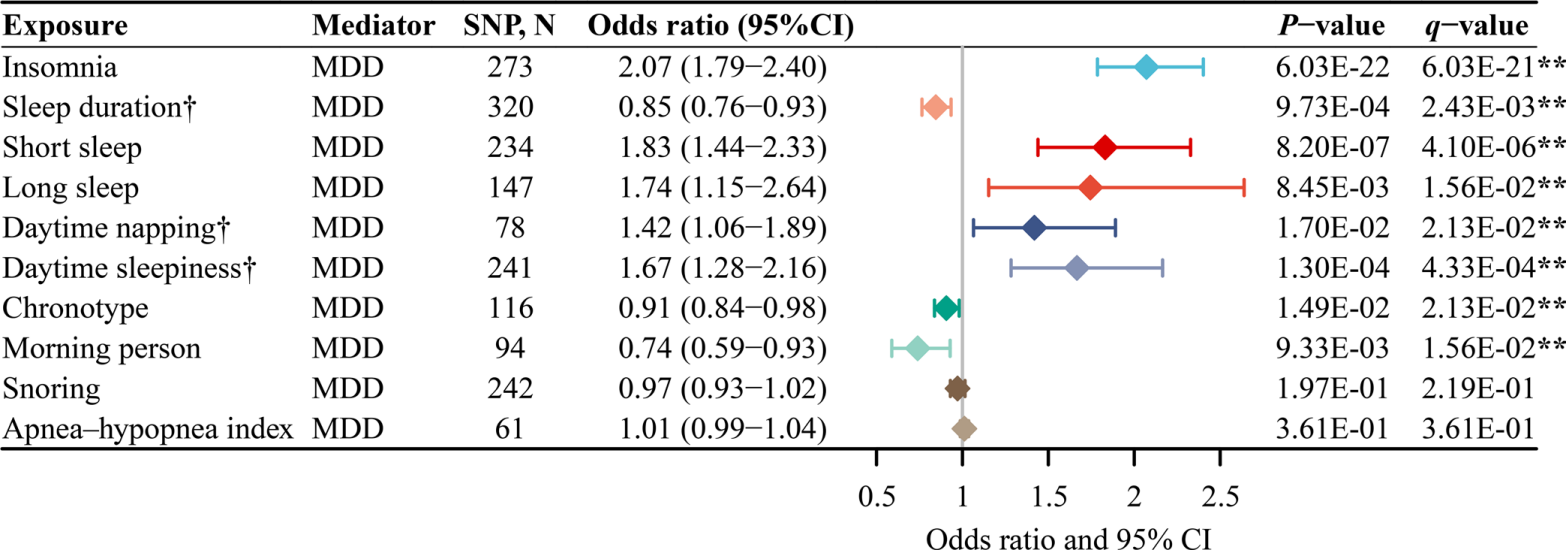
The main MR analysis results of the causal effects of sleep traits on MDD. The OR represented the effect of genetically predicted per unit increase in each sleep trait. **Significant evidence (q-value<0.05) after correction for multiple testing estimated by the FDR method. †Because there was significant heterogeneity, the random-effect IVW model was used, while for others, because there was no significant heterogeneity, the fixed-effect IVW model was used. FDR, false discovery rate; IVW, inverse-variance weighted; MDD, major depressive disorder; MR, Mendelian randomisation; SNP, single-nucleotide polymorphism.

The MR-Egger intercept test confirmed the absence of significant horizontal pleiotropy (p>0.05) (online supplemental table 6). Figure 3 illustrates that genetically predicted insomnia (OR 2.07 (95% CI 1.79 to 2.40)), short sleep (OR 1.83 (95% CI 1.44 to 2.33)), long sleep (OR 1.74 (95% CI 1.15 to 2.64)), daytime napping (OR 1.42 (95% CI 1.06 to 1.89)) and daytime sleepiness (OR 1.67 (95% CI 1.28 to 2.16)) were associated with a significantly increased risk of MDD. Conversely, genetically predicted sleep duration (OR 0.85 (95% CI 0.76 to 0.93)), chronotype (OR 0.91 (95% CI 0.84 to 0.98)) and being a morning person (OR 0.74 (95% CI 0.59 to 0.93)) were associated with a significantly decreased risk of MDD.

### The effect of MDD on the risk of each CVD after adjusting each sleep trait

Online supplemental table 7 provides results of the causal effect of MDD on the risk of each CVD without adjustment for any sleep traits. Online supplemental figure 2 and table 8 present results of the causal effect of MDD on the risk of each CVD after adjusting for each sleep trait, showing that genetically predicted per log odds increase in MDD still significantly increased the risks of CAD, MI, HF and stroke, and the OR values ranged from 1.04 to 1.08. However, there was no evidence that MDD increased the risk of AF, with the q-values for FDR ranging from 0.207 for adjusting daytime sleepiness to 0.751 for adjusting long sleep.

### Mediation effect of MDD

Table 2 shows that genetically predicted MDD mediates the effect of sleep traits on the risk of CVDs. The effect of insomnia was partially mediated by MDD on the risk of CAD (proportion mediated 8.81% (95% CI 1.20% to 16.43%)), MI (proportion mediated 9.17% (95% CI 1.71% to 16.63%)) and HF (proportion mediated 14.46% (95% CI 3.48% to 25.45%)). The effect of short sleep duration was partially mediated by MDD on the risk of CAD (proportion mediated 8.92% (95% CI 0.87% to 16.97%)), MI (proportion mediated 11.43% (95% CI 0.28% to 22.57%)) and HF (proportion mediated 12.65% (95% CI 1.35% to 23.96%)). In addition, we also found that although the mediation proportion of MDD did not reach statistical significance in the effects of insomnia on stroke, sleep duration on CAD, MI and HF, daytime napping on HF and daytime sleepiness on CAD, the mediation effect of MDD in the foregoing associations was significant, which indicated that MDD might act as a mediator in the foregoing causal pathway.

**Table 2:** The significantly estimated proportion mediated for the effect of sleep traits on CVDs explained by MDD

Exposure	Outcome	Total effect: βc (95% CI)*	Direct effect: βa (95% CI)†	Direct effect: βb (95% CI)‡	Mediation effect(95% CI)§	P value¶	Proportion mediated,% (95% CI)**
Insomnia	CAD	0.38(0.21, 0.56)	0.73(0.58, 0.88)	0.05(0.01, 0.08)	0.034(0.007, 0.061)	0.015	**8.81 (1.20, 16.43**)
Insomnia	MI	0.44(0.24, 0.63)	0.73(0.58, 0.88)	0.06(0.02, 0.10)	0.040(0.010, 0.071)	0.009	**9.17 (1.71, 16.63**)
Insomnia	HF	0.27(0.13, 0.42)	0.73(0.58, 0.88)	0.05(0.02, 0.09)	0.039(0.014, 0.064)	0.002	**14.46 (3.48, 25.45**)
Insomnia	Stroke	0.23(0.03, 0.43)	0.73(0.58, 0.88)	0.04(0.00, 0.08)	0.030(0.001, 0.058)	0.040	13.09 (−2.69, 28.86)
Sleep duration	CAD	−0.12(−0.22, –0.03)	−0.17(−0.27, –0.07)	0.06(0.02, 0.09)	−0.01(−0.018, –0.001)	0.021	8.00 (−0.78, 16.78)
Sleep duration	MI	−0.11(−0.22, –0.01)	−0.17(−0.27, –0.07)	0.07(0.03, 0.11)	−0.012(−0.021, –0.002)	0.017	10.22 (−1.87, 22.32)
Sleep duration	HF	−0.10(−0.18, –0.02)	−0.17(−0.27, –0.07)	0.06(0.04, 0.09)	−0.011(−0.019, –0.003)	0.009	10.70 (−0.30, 21.70)
Short sleep	CAD	0.44(0.15, 0.72)	0.60(0.36, 0.84)	0.06(0.03, 0.10)	0.039(0.012, 0.066)	0.005	**8.92 (0.87, 16.97**)
Short sleep	MI	0.40(0.09, 0.72)	0.60(0.36, 0.84)	0.08(0.04, 0.12)	0.046(0.016, 0.077)	0.003	**11.43 (0.28, 22.57**)
Short sleep	HF	0.33(0.09, 0.56)	0.60(0.36, 0.84)	0.07(0.04, 0.10)	0.041(0.016, 0.066)	0.001	**12.65 (1.35, 23.96**)
Daytime napping	HF	0.33(0.11, 0.55)	0.35(0.06, 0.64)	0.06(0.03, 0.09)	0.022(0.000, 0.043)	0.045	6.52 (−0.86, 13.90)
Daytime sleepiness	CAD	0.33(0.08, 0.59)	0.51(0.25, 0.77)	0.05(0.01, 0.08)	0.025(0.003, 0.047)	0.024	7.63 (−0.84, 16.10)

* Total effect βc: the effect of each sleep trait on the risk of CVDs.

† Direct effect βa: the effect of each sleep trait on the risk of MDD.

‡ Direct effect βb: the effect of MDD on the risk of CVDs after adjusting each sleep trait.

§ Mediation effect: the effect of each sleep trait on the CVDs acting through MDD. The 95% CI CI of the mediation effect is calculated by the following formula: $\beta_{a}\times \beta_{b}\pm 1.96\times \sqrt{\beta_{b}^{2}\times {se}_{a}^{2}+\beta_{a}^{2}\times {se}_{b}^{2}}$, where ${se}_{a}$denotes the standard errorSE of $\beta_{a}$, ${se}_{b}$denotes the standard errorSE of $\beta_{b}$.

¶ P-v value refers to the result of the Sobel test for mediation effect.

** Boldface type indicates statistically significant findings. The mediation proportion was interpreted as statistically significant if the 95% CI CI of the percent mediated effect did not include the null value.

CAD, coronary artery disease; CVDscardiovascular diseasesHF, heart failureMDD, major depressive disorder; MI, myocardial infarction

### Sensitivity analyses

We further verified the robustness of the results through a variety of supplementary MR analyses (online supplemental tables 9–11). The results showed that the maximum likelihood method, IVW-SIMEX and median method (including weighted median and penalised weighted median) were consistent with the main analysis results. The effect size and direction of the MR Egger method were also consistent, although some results may not be statistically significant due to the lower precision of the MR Egger method. The results of the radial IVW method (online supplemental tables 12–14) and replicate MR analyses under strict thresholds (online supplemental tables 15–17) were consistent with the effect size and direction of main analysis results, but some may not be statistically significant due to lower statistical power. The leave-one-out analysis indicated that no single SNP strongly influenced the results of causal effects. In multivariable MR analyses, the effect size and direction of multivariable IVW, MR Egger and QHET methods were also consistent (online supplemental table 18).

## Discussion

This study represents the most extensive MR analysis investigation to date, delving into the causal relationships between sleep traits, MDD and CVDs. Notably, it pioneers the exploration of MDD’s potential mediating role in the nexus between sleep traits and CVDs. Our findings offer compelling genetic evidence: insomnia is linked to heightened risks of MDD and all five CVDs examined (CAD, MI, HF, AF and stroke); meanwhile, sleep duration correlates with decreased risks of MDD and three CVDs (CAD, MI and HF), with short sleep emerging as a risk factor for these conditions. Additionally, long sleep solely associates with increased MDD risk, whereas chronotype and being a morning person inversely correlate with MDD risk. Furthermore, frequent daytime napping is associated with heightened risks of MDD, HF and AF, while frequent daytime sleepiness elevates risks of MDD and CAD. Our mediation analysis reveals that MDD mediates between 8.81% and 14.46% of the effects of insomnia and short sleep on CAD, MI and HF.

Although previous observational analyses yielded inconsistent results,^5 31^ our findings align with three meta-analyses^32^,^34^ and two MR analyses,^11 35^ all indicating that insomnia escalates the risk of CVDs. Moreover, our study uniquely supplements this literature by revealing that insomnia is also associated with an increased risk of MI, a subtype of CVDs. Interestingly, Liu *et al*^11^ reported that the causal effect of insomnia on the risk of CVDs was mediated by 17 cardiometabolic risk factors (excluding MDD). In contrast, our research underscores the substantial mediation effect of MDD (8.81% to 14.46%), second only to BMI (14.94% to 29.16%) in their study. This suggests a critical focus on depression prevention among individuals experiencing frequent insomnia to mitigate CVD risks.

A prospective study of 116 632 people from 21 countries^3^ reported that short sleep duration was not associated with an increased risk of CVDs. However, two meta-studies by Watanabe *et al*^36 37^ reported that both long and short sleep duration increased the risk of CVDs. These may lead to contradictory results due to the limitations of observational studies. The MR study conducted by Zhuang *et al*^38^ failed to establish a causal relationship between sleep duration and the risk of CAD and MI, which may be due to the bias caused by IVs of exposure from a small sample. Furthermore, our study underscores the increased risk of MDD, CAD, MI and HF associated with short sleep, along with the heightened MDD risk linked to long sleep. We advocate for maintaining a consistent nightly sleep duration within the normal range per day for optimal health.

Currently, there is a scarcity of MR studies examining the association between daytime napping, sleepiness frequency and the risk of CVDs. Our MR investigation fills this gap by revealing that increased daytime napping frequency heightens the risk of HF and AF, while elevated daytime sleepiness frequency correlates with a heightened risk of CAD. Moreover, our conclusions find support from underlying physiological mechanisms, as evidenced by MR findings indicating potential causal links between more frequent daytime napping and elevated blood pressure and waist circumference.^17^ Additionally, genetic evidence underscores associations between daytime napping, sleepiness frequency and obesity,^39^ further reinforcing our study’s findings.

Currently, some studies have defined the low-risk group for CVDs according to five sleep types including early chronotype, sleep 7–8 hours per day, never/rarely experience insomnia, no snoring and no frequent excessive daytime sleepiness.^4^ However, we did not find directly significant protective effects of chronotype, morning person and no snoring on CVDs risk at the genetic level. It should be added that we still recommend chronotype and morning person as they have a protective effect on the risk of MDD, which is a risk factor for CVDs. Notably, the conclusions drawn by Jia *et al*,^10^ suggesting that being a morning person may be a potential ‘risk factor’ for cardiometabolic diseases, under the violation of the MR assumptions need to be interpreted with caution. Although there may be a bidirectional causal relationship between MDD and CVDs, the assessment and prevention of MDD after CVDs diagnosis has been emphasised by doctors and patients. Therefore, our study focused more on further evaluating the effect of MDD as a psychological factor on the risk of CVDs. Our study also suggests that MDD may mediate the relationship between insomnia and CVD, highlighting the importance of early detection and treatment of MDD in patients with insomnia and short sleep duration. Therefore, future CVDs prevention and early screening policies should pay extra attention to poor sleep traits (insomnia, short sleep, frequent daytime naps and sleepiness) and MDD, which will have important public health significance.

There are several limitations to this study. First, although most of the sleep traits in our study were self-reported rather than objectively measured, self-reported data may be more suitable for assessing long-term sleep patterns in large-scale studies. Second, an inherent limitation of the MR analysis is that there may be potential polymorphic effects, therefore, we have removed SNPs associated with potential confounding and those with pleiotropic outliers detected by the MR-PRESSO method. The MR Egger intercept test was used to estimate pleiotropy to prevent bias caused by pleiotropy as much as possible. Third, although the GWAS data of the apnoea-hypopnoea index included is the largest to date, the sample size (n=5727 Europeans) is still relatively small, so larger GWAS data may be needed for further validation. Fourth, the MDD GWAS data used in this study included a small subset of UK Biobank participants, which can be removed for further validation as future data becomes available. Finally, due to the availability of data, this study mainly focused on the population of European descent. In the future, with the development of large-scale GWAS, further research on other populations will help to confirm and support our findings reported here.

## Conclusions

In summary, evidence from our MR study suggests that genetically predicted insomnia, short sleep and frequent daytime napping or sleepiness can increase the risk of certain CVDs and MDD, and being a morning person can reduce the risk of MDD. Moreover, our study provides genetic evidence that the causal effect of sleep traits on CVDs is partly mediated by MDD. The foregoing findings support the increased consideration of sleep traits and MDD in the prevention and screening policies for CVDs.

## supplementary material

10.1136/openhrt-2024-002866 online supplemental table 1

## Data Availability

Data are available in a public, open access repository.
