# Supplementary material for: Investigating the impact of multidimensional sleep traits on cardiovascular diseases and the mediating role of depression
Source: Open Heart. 2025 Mar 13;12(1):e002866. doi: 10.1136/openhrt-2024-002866 (PMC11907084; doi:10.1136/openhrt-2024-002866)
Supplement: online supplemental table 1 [file openhrt-12-1-s001.docx]

**Supplementary material**

**Index**

eMethod 1. Supplemental methods for GWAS data sources of exposure.………………………………………………. 1

eMethod 2. Selection of genetic instruments and MR analysis procedure.....……………………....………….…….......3

eMethod 3. Supplemental methods for mediation analyses of Mendelian randomization study....……. ……………......5

[Table S1. Screening process of final SNPs of instrumental variables for each MR analysis. 6](#_Toc183116667)

[Table S2. Details of removed SNPs associated with confounders. 9](#_Toc183116668)

[Table S3. Details of removed SNPs (missing, ambiguous palindromic, and outlier). 21](#_Toc183116669)

[Table S4. The priori statistical power in the MR analysis of the causal effects. 25](#_Toc183116670)

[Table S5. The pleiotropy and heterogeneity for primary univariate MR analyses of sleep traits on CVDs. 27](#_Toc183116671)

[Table S6. The pleiotropy and heterogeneity for primary univariate MR analyses of sleep traits on MDD. 28](#_Toc183116672)

[Table S7. The main univariate MR analyses results of the causal effects of MDD on CVDs. 29](#_Toc183116673)

[Table S8. The pleiotropy and heterogeneity test results of effects of MDD on each CVDs after adjusting for each sleep trait. 29](#_Toc183116674)

[Table S9. The complementary MR analysis results of the causal effects of sleep traits on CVDs. 31](#_Toc183116675)

[Table S10. The complementary MR analysis results of the causal effects of sleep traits on MDD. 32](#_Toc183116676)

[Table S11. The complementary MR analysis results of the causal effects of MDD on CVDs. 33](#_Toc183116677)

[Table S12. Verification results of potentially weak instrumental bias in causal effects of sleep traits on CVDs. 34](#_Toc183116678)

[Table S13. Verification results of potentially weak instrumental bias in causal effects of sleep traits on MDD. 35](#_Toc183116679)

[Table S14. Verification results of potentially weak instrumental bias in causal effects of MDD on CVDs. 35](#_Toc183116680)

[Table S15. Replicate MR analyses results of the effect of sleep traits on CVDs screened by strict threshold criteria. 35](#_Toc183116681)

[Table S16. Replicate MR analyses results of the effect of sleep traits on MDD screened by strict threshold criteria. 36](#_Toc183116682)

[Table S17. Replicate MR analyses results of the effect of MDD on CVDs screened by strict threshold criteria. 36](#_Toc183116683)

[Table S18. The multiple multivariable MR analyses results of effects of MDD on each CVDs after adjusting for each sleep trait. 37](#_Toc183116684)

[Figure S1. The assumption of MR model. 39](#_Toc183116685)

[Figure S2. The main MR analysis results of the causal effects of MDD on CVDs after adjusting for each sleep trait. 40](#_Toc183116686)

## eMethod 1. Supplemental methods for GWAS data sources for exposure.

### Insomnia

To assess insomnia symptoms, participants in UK Biobank (UKB) cohort of European ancestry were asked: “Do you have trouble falling asleep at night or do you wake up in the middle of the night?” with responses “never/rarely” (n = 108,357), “sometimes” (n = 215,752), “usually” (n = 129,270), “prefer not to answer” (n = 637). Those responded “prefer not to answer” were set to missing. Participants with usually insomnia were defined as cases of frequent insomnia symptoms, and participants with never/rarely insomnia were defined as controls, that is, frequent insomnia symptoms (“never/rarely” vs. “usually” insomnia symptoms, 129,270 cases and 108,357 controls).^1^

### Sleep duration, short sleep, and long sleep

To assess sleep duration, participants in UKB cohort of European ancestry were asked: “About how many hours sleep do you get in every 24 h? (please include naps)”, with responses in hour increments. Sleep duration (n =  446,118) was treated as a continuous variable and also categorized as short (≤6 h per night; n = 106,192 cases) and long (≥9 h per night; n = 34,184 cases) sleep relative to normal (7-8 h per night; n = 305,742 controls).^2^

### Daytime napping

To assess daytime napping frequency, participants in UKB cohort of European ancestry were asked: “Do you have a nap during the day?”, with responses “never/rarely” (n = 255,746), “sometimes” (n = 172,897), “usually” (n = 23,990), “prefer not to answer” (as missing; n = 49,013). Daytime napping frequency (n = 452,633) was treated as an ordered categorical variable.^3^

### Daytime sleepiness

To assess the frequency of daytime sleepiness, participants in UKB cohort of European ancestry were asked: “How likely are you to doze off or fall asleep during the daytime when you don’t mean to? (e.g.: when working, reading or driving)”, with the answer categories “never” (n = 347,285), “sometimes” (n = 92,794), “often” (n = 11,963), or “all of the time” (n = 29). Daytime sleepiness frequency (n = 452,071) was treated as an ordered categorical variable.^4^

### Chronotype and morning person

To assess chronotype (“Morning/evening person”), participants in UKB cohort of European ancestry were asked: “Do you consider yourself to be?” with one of six possible answers: “definitely a ‘morning’ person” (n= 107,555), “more a ‘morning’ than ‘evening’ person” (n = 144,731), “more an ‘evening’ than a ‘morning’ person” (n = 115,090), “definitely an ‘evening’ person” (n = 35,818), “do not know” (n = 46,540), which we coded as 2, 1, −1, −2, 0, respectively. Chronotype based on phenotype coding (n = 449,734) were treated as an ordered categorical variable in the GWAS. Morning person also defined a binary variable by classifying the morning preference (Coded 2 and 1; n = 252,287 cases) and the evening preference (Coded -2 and -1; n = 150,908 controls).^5^

### Snoring

To assess snoring, participants (n = 359,916) in UKB cohort of European ancestry were asked: “Does your partner or a close relative or friend complain about your snoring?”, with responses “yes” (37.3%) or “no” (62.7%).^6^

### Obstructive sleep apnea

Obstructive sleep apnea (OSA) was quantified using the apnea–hypopnea index (AHI), defined as the number of apnea and hypopnea events per hour of sleep. The AHI related GWAS data were obtained from 5,727 European Americans in five cohorts, including: the ARIC (Atherosclerosis Risk in Communities Study; n = 1,463), the CFS (Cleveland Family Study; n = 702), the FHS (Framingham Heart Study; n = 646), the MESA (Multi-Ethnic Study of Atherosclerosis; n = 707), and the MrOS (Osteoporotic Fractures in Men Study; n = 2,209).^7^

## eMethod 2. Selection of genetic instruments and sensitivity analyses.

The SNPs of IVs were screened and determined by the following steps. First, SNPs associated with each trait at genome-wide significance (*P* <5e-8) were extracted, and then independent SNPs were obtained by clustering with linkage disequilibrium (LD) threshold r^2^ <0.001 and distance of 10,000 kb. To further instrument power of MR analyses, when the explained variance of IVs under the strict threshold was less than 1%, we relaxed the significance threshold to *P* <5e-5 to increase the explained variance of IVs as much as possible and further meet the statistical power of MR analyses, which has been widely used in previous studies.^8-10^ Second, we removed SNPs associated with potential confounders with a correlation threshold of 1e-5 by the web-interfaced PhenoScanner (a curated database of publicly available GWAS).^11^ These confounding factors included obesity, blood lipids, blood glucose, blood pressure, physical activity, education, smoking, and drinking. Third, SNPs for IVs of exposure were extracted from each outcome GWAS. When SNPs for IVs of exposure were not available in the outcome GWAS, the LDlink tool ^12^ was used to identify proxy SNPs of European ancestry (r^2^ >0.8). Fourth, SNPs in IVs of exposure and outcome data were harmonized, and palindromic SNPs were inferred based on the effect allele frequency (EAF) by setting action = 2 (If the EAF of palindromic SNPs was incomplete, the SNPs with 0.4 < EAF < 0.6 were regarded as ambiguous palindromes and removed.).^13^ Finally, MR Pleiotropy RESidual Sum and Outlier (MR-PRESSO) was used to further detect SNPs of potential horizontal pleiotropic outliers, and where significant outliers (*P* <0.05) were detected, they were removed from the following analyses to obtain an unbiased causal estimate.^14^ The final IVs were obtained through the above series of processes and the explained variance was reassessed. The F-statistics of >10 for each SNP indicated a relatively low risk of weak instrument bias.^8^ The priori statistical power was calculated using the mRnd power calculation online tool.^15^

To further assess the robustness of our findings, a series of sensitivity analyses were performed. First, complementary MR analyses, including likelihood-based MR,^16^ MR-Egger,^17^ IVW-SIMEX (simulation extrapolation),^18^ weighted median, and penalized weighted median^19^ were applied to help verify causal inference in the presence of a small proportion of invalid instruments or pleiotropy bias. Second, to further verify the possible weak instrumental bias caused by relaxing the threshold (*P* <5e-5), we used Radial IVW method,^20^ and re-performed MR analyses under strict threshold (*P* <5e-8). Finally, the leave-one-out sensitivity analysis was conducted by removing a single SNP from the analysis in turn to assess the influence of individual variants on the causal estimation.

## eMethod 3. Supplemental methods for mediation analyses of Mendelian randomization study.

First, to estimate mediation effect ($\mathrm{ME}$, i.e., indirect effect), we estimated the effect ($\beta_{a}$) of each exposure (i.e., sleep traits) on the mediator (i.e., MDD) individually using univariable MR analyses, then we multiplying this with the effect ($\beta_{b}$) of that the mediator (i.e., MDD) on each outcome (i.e., CVDs) after adjusting for each exposure (i.e., sleep traits) using multivariable MR analyses ^21^. The ME formula is as follows:

$$\begin{aligned} Mediation effect:ME=\beta_{a}\times\beta_{b}\#\left( 1 \right) \end{aligned}$$

where $\beta_{a}$ is the effect of exposure on mediator, and $\beta_{b}$ is the effect of mediator on outcome after adjusting exposure. The calculation of standard error of ME is based on Sobel test ^22^ by the following formula:

$$\begin{aligned} Standard error of ME:{se}_{ME}^{2}= \beta_{b}^{2}\times{se}_{a}^{2}+\beta_{a}^{2}\times{se}_{b}^{2}\#\left( 2 \right) \end{aligned}$$

where ${se}_{a}$ denotes the standard error of $\beta_{a}$, and ${se}_{b}$denotes the standard error of $\beta_{b}$. The 95% CI of ME by the following formula:

$$\begin{aligned} The 95\% CI of ME: ME\pm1.96\times{se}_{ME}\#\left( 3 \right) \end{aligned}$$

where ME is the mediation effect, and ${se}_{ME}$ denotes the standard error of ME.

Finally, we divided the ME by the total effect ($\beta_{c}$) of each exposure (i.e., sleep traits) on each outcome (i.e., CVDs) to estimate the mediation proportion (MP). The MP formula is as follows:

$$\begin{aligned} \mathrm{Mediation}\mathrm{proportion}:MP=\frac{ME}{\beta_{c}}\#\left( 4 \right) \end{aligned}$$

where $\beta_{c}$ is the total effect of exposure on outcome. The calculation of standard error of MP is based on the Delta method^23^ by the following formula:

$$\begin{aligned} Standard error of MP:{se}_{MP}^{2}= \frac{{se}_{ME}^{2}}{\beta_{c}^{2}}+\frac{{ME}^{2}\times{se}_{c}^{2}}{\beta_{c}^{4}}-2\times\frac{ME\times{se}_{ME}^{2}}{\beta_{c}^{3}}\#\left( 5 \right) \end{aligned}$$

where ${se}_{c}$ denotes the standard error of $\beta_{c}$. The 95% CI of MP by the following formula:

$$\begin{aligned} The 95\% CI of MP: MP\pm1.96\times{se}_{MP}\#\left( 6 \right) \end{aligned}$$

where MP is the mediation proportion, and ${se}_{MP}$ denotes the standard error of MP.

## Table S1. Screening process of final SNPs of instrumental variables for each MR analysis.

| Exposure/Mediator* | Mediator/ Outcome† | Number of candidate SNPs, N | Remove SNPs associated with confounders, N‡ | Remove SNPs missing in the outcome, N§ | Remove ambiguous palindromic SNPs, N§ | Remove outlier SNPs, N§ | Number of final SNPs, N |
| --- | --- | --- | --- | --- | --- | --- | --- |
| Insomnia | MDD | 337 | 50 | 0 | 10 | 4 | 273 |
|  | CAD | 337 | 50 | 8 | 9 | 0 | 270 |
|  | MI | 337 | 50 | 10 | 9 | 0 | 268 |
|  | HF | 337 | 50 | 10 | 10 | 0 | 267 |
|  | AF | 337 | 50 | 0 | 10 | 0 | 277 |
|  | Stroke | 337 | 50 | 6 | 10 | 0 | 271 |
| Sleep duration | MDD | 392 | 57 | 1 | 11 | 3 | 320 |
|  | CAD | 392 | 57 | 6 | 11 | 1 | 317 |
|  | MI | 392 | 57 | 6 | 11 | 0 | 318 |
|  | HF | 392 | 57 | 9 | 12 | 0 | 314 |
|  | AF | 392 | 57 | 1 | 13 | 6 | 315 |
|  | Stroke | 392 | 57 | 7 | 12 | 1 | 315 |
| Short sleep | MDD | 285 | 41 | 0 | 8 | 2 | 234 |
|  | CAD | 285 | 41 | 5 | 11 | 0 | 228 |
|  | MI | 285 | 41 | 6 | 12 | 0 | 226 |
|  | HF | 285 | 41 | 9 | 8 | 0 | 227 |
|  | AF | 285 | 41 | 0 | 13 | 2 | 229 |
|  | Stroke | 285 | 41 | 5 | 10 | 0 | 229 |
| Long sleep | MDD | 174 | 23 | 3 | 1 | 0 | 147 |
|  | CAD | 174 | 23 | 8 | 1 | 0 | 142 |
|  | MI | 174 | 23 | 10 | 1 | 0 | 140 |
|  | HF | 174 | 23 | 13 | 1 | 1 | 136 |
|  | AF | 174 | 23 | 5 | 1 | 2 | 143 |
|  | Stroke | 174 | 23 | 8 | 1 | 1 | 141 |
| Daytime napping^#^ | MDD | 104 | 21 | 0 | 2 | 3 | 78 |
|  | CAD | 104 | 21 | 0 | 2 | 0 | 81 |
|  | MI | 104 | 21 | 0 | 2 | 0 | 81 |
|  | HF | 104 | 21 | 0 | 2 | 0 | 81 |
|  | AF | 104 | 21 | 1 | 2 | 1 | 79 |
|  | Stroke | 104 | 21 | 0 | 2 | 0 | 81 |
| Daytime sleepiness | MDD | 286 | 38 | 2 | 4 | 1 | 241 |
|  | CAD | 286 | 38 | 8 | 6 | 0 | 234 |
|  | MI | 286 | 38 | 9 | 5 | 0 | 234 |
|  | HF | 286 | 38 | 15 | 4 | 0 | 229 |
|  | AF | 286 | 38 | 3 | 6 | 1 | 238 |
|  | Stroke | 286 | 38 | 9 | 4 | 0 | 235 |
| Chronotype^#^ | MDD | 152 | 35 | 0 | 1 | 0 | 116 |
|  | CAD | 152 | 35 | 1 | 1 | 0 | 115 |
|  | MI | 152 | 35 | 1 | 1 | 0 | 115 |
|  | HF | 152 | 35 | 0 | 1 | 0 | 116 |
|  | AF | 152 | 35 | 0 | 1 | 0 | 116 |
|  | Stroke | 152 | 35 | 0 | 1 | 1 | 115 |
| Morning person^#^ | MDD | 122 | 25 | 0 | 3 | 0 | 94 |
|  | CAD | 122 | 25 | 1 | 3 | 0 | 93 |
|  | MI | 122 | 25 | 1 | 3 | 0 | 93 |
|  | HF | 122 | 25 | 0 | 3 | 0 | 94 |
|  | AF | 122 | 25 | 0 | 3 | 1 | 93 |
|  | Stroke | 122 | 25 | 0 | 3 | 1 | 93 |
| Snoring | MDD | 294 | 47 | 0 | 3 | 2 | 242 |
|  | CAD | 294 | 47 | 1 | 4 | 0 | 242 |
|  | MI | 294 | 47 | 3 | 6 | 1 | 237 |
|  | HF | 294 | 47 | 3 | 2 | 1 | 241 |
|  | AF | 294 | 47 | 0 | 6 | 6 | 235 |
|  | Stroke | 294 | 47 | 0 | 2 | 0 | 245 |
| Apnea–hypopnea index | MDD | 65 | 1 | 0 | 3 | 0 | 61 |
|  | CAD | 65 | 1 | 1 | 2 | 0 | 61 |
|  | MI | 65 | 1 | 2 | 2 | 0 | 60 |
|  | HF | 65 | 1 | 3 | 3 | 0 | 58 |
|  | AF | 65 | 1 | 1 | 3 | 0 | 60 |
|  | Stroke | 65 | 1 | 2 | 3 | 0 | 59 |
| MDD | CAD | 207 | 16 | 7 | 3 | 0 | 181 |
|  | MI | 207 | 16 | 9 | 4 | 0 | 178 |
|  | HF | 207 | 16 | 12 | 3 | 0 | 176 |
|  | AF | 207 | 16 | 5 | 4 | 0 | 182 |
|  | Stroke | 207 | 16 | 4 | 3 | 0 | 184 |

MDD, major depressive disorder; CAD, coronary artery disease; MI, myocardial infarction; HF, heart failure; AF, atrial fibrillation.

*Exposure includes insomnia, sleep duration, long sleep, daytime napping, daytime sleepiness, chronotype, morning person, snoring, and apnea–hypopnea index. Mediator means MDD.

†Mediator means MDD. Outcome includes CAD, MI, HF, AF, and Stroke.

#IVs were screened by strict threshold standard (significant *P* <5e-8), and others were relaxed threshold standards (suggestive *P* <5e-5). Independent SNPs under the two sets of threshold standards were obtained by clustering with linkage disequilibrium (LD) threshold r^2^ < 0.001 and distance of 10,000kb.

‡Detailed SNPs associated with confounders are shown in Supplementary Table 3

§Detailed SNPs (missing, ambiguous palindromic, and outlier) are shown in Supplementary Table 4

## Table S2. Details of removed SNPs associated with confounders.

| Exposure/Mediator (N)* | SNPs | *P*-value association with confounders† | | | | | | | |
| --- | --- | --- | --- | --- | --- | --- | --- | --- | --- |
|  |  | Obesity | Blood lipids | Blood glucose | Blood pressure | Physical activity | Education | Smoking | Drinking |
| Insomnia (50) | rs1008078 | 5.13E-08 |  |  |  | 1.34E-06 | 7.88E-11 | 4.18E-06 |  |
|  | rs10280045 |  |  |  |  | 1.29E-11 |  |  |  |
|  | rs10838708 |  | 1.55E-10 |  |  |  |  |  |  |
|  | rs10891492 |  |  |  | 3.27E-06 |  |  | 6.07E-36 |  |
|  | rs11097861 |  |  |  |  |  |  | 4.18E-06 |  |
|  | rs11635495 | 5.58E-11 |  |  | 2.02E-06 |  |  | 5.83E-08 |  |
|  | rs11793074 |  |  |  |  |  |  | 2.14E-07 |  |
|  | rs12551649 |  |  |  |  |  |  |  | 2.14E-06 |
|  | rs12721051 | 1.12E-11 | 3.23E-24 | 3.30E-08 |  | 1.46E-07 |  |  |  |
|  | rs13103161 |  |  |  |  | 1.69E-10 | 1.51E-06 |  | 2.07E-07 |
|  | rs13215045 | 9.15E-07 |  |  |  |  |  |  |  |
|  | rs1430205 |  |  |  |  |  |  |  | 7.50E-09 |
|  | rs1542212 | 2.10E-07 |  |  |  |  |  |  |  |
|  | rs1592757 |  |  |  |  |  | 5.71E-06 | 6.15E-06 |  |
|  | rs1599337 | 2.73E-19 |  |  | 3.04E-06 |  |  | 1.21E-06 |  |
|  | rs16939357 |  |  |  | 8.31E-09 |  |  |  |  |
|  | rs17151854 |  |  |  | 6.40E-06 |  |  |  |  |
|  | rs1974620 |  |  | 9.00E-07 |  |  |  |  |  |
|  | rs2045458 | 1.00E-06 | 6.40E-06 |  |  |  |  |  | 7.80E-10 |
|  | rs2075678 |  |  |  |  |  | 9.50E-08 |  |  |
|  | rs224071 |  |  |  | 4.62E-08 |  |  |  |  |
|  | rs2297787 | 4.77E-07 |  |  | 1.57E-13 |  |  |  |  |
|  | rs2487512 | 2.61E-12 |  |  |  |  |  |  |  |
|  | rs2501279 | 9.82E-06 |  |  |  |  |  |  |  |
|  | rs2602205 |  |  |  | 4.50E-06 |  |  |  |  |
|  | rs2644128 | 2.05E-18 |  |  | 1.80E-07 |  |  |  |  |
|  | rs2736209 | 7.16E-10 |  |  |  |  |  |  |  |
|  | rs2838788 | 1.58E-07 |  |  |  |  |  |  |  |
|  | rs34584592 | 2.10E-07 |  |  |  |  |  |  |  |
|  | rs35713119 | 3.95E-07 |  |  |  |  |  |  |  |
|  | rs41271299 | 9.99E-10 |  |  |  |  |  |  |  |
|  | rs4352361 |  |  | 5.28E-07 |  |  |  |  |  |
|  | rs4688760 | 5.38E-21 |  | 5.27E-09 | 3.64E-06 |  |  |  | 5.67E-06 |
|  | rs4751 | 1.23E-08 |  |  | 4.66E-07 |  |  |  |  |
|  | rs4886860 | 3.61E-06 |  |  |  |  |  |  |  |
|  | rs524859 | 7.10E-10 |  |  |  |  |  |  | 1.34E-06 |
|  | rs55670730 | 1.09E-14 |  |  | 8.02E-07 |  |  |  |  |
|  | rs57272144 |  |  |  |  |  |  | 1.21E-07 |  |
|  | rs6141314 |  |  |  |  |  |  | 7.51E-06 |  |
|  | rs641325 |  |  |  | 5.42E-06 | 5.29E-08 |  |  | 2.22E-06 |
|  | rs6478530 | 4.81E-06 |  |  |  |  | 2.10E-06 |  | 7.51E-06 |
|  | rs66833742 | 8.59E-15 |  |  |  |  |  |  |  |
|  | rs67007480 | 1.28E-06 |  |  |  |  |  |  |  |
|  | rs72783611 |  |  |  |  |  |  |  | 4.94E-08 |
|  | rs7572387 | 2.95E-18 |  |  |  |  |  |  |  |
|  | rs76411246 | 7.27E-06 |  |  |  |  |  |  |  |
|  | rs7781195 |  |  |  |  | 3.08E-06 |  |  | 8.19E-06 |
|  | rs79589176 | 6.06E-09 |  |  |  |  |  |  |  |
|  | rs9466936 | 1.98E-06 |  |  |  |  |  |  |  |
|  | rs9894577 |  |  |  | 6.65E-08 |  |  |  |  |
| Sleep duration (57) | rs10116103 |  |  |  |  |  | 3.51E-11 |  |  |
|  | rs10145592 |  |  |  |  |  | 1.10E-06 |  |  |
|  | rs10264127 | 6.97E-06 |  |  |  |  |  |  |  |
|  | rs10421649 |  |  |  |  |  | 5.72E-06 |  |  |
|  | rs10483350 | 1.08E-06 |  |  |  |  |  |  |  |
|  | rs10761674 |  |  |  | 1.90E-08 |  |  |  |  |
|  | rs11135570 |  | 6.75E-08 |  |  |  |  |  |  |
|  | rs11186923 |  |  | 3.20E-06 |  |  |  |  |  |
|  | rs113113059 |  |  |  |  | 4.72E-06 |  |  |  |
|  | rs115319635 | 1.33E-07 |  |  |  |  |  |  |  |
|  | rs11689042 | 1.11E-12 |  |  |  |  |  |  |  |
|  | rs11695684 |  |  |  |  |  |  | 9.02E-06 |  |
|  | rs11928693 | 3.70E-06 |  |  |  |  |  |  |  |
|  | rs12030413 |  |  |  |  |  |  |  | 6.70E-08 |
|  | rs12246842 | 3.14E-09 |  |  |  |  |  |  |  |
|  | rs1252228 | 2.58E-06 |  |  |  |  |  |  |  |
|  | rs12598706 | 6.80E-06 |  |  | 4.06E-06 |  |  |  |  |
|  | rs12760654 | 7.87E-07 |  |  |  |  |  |  |  |
|  | rs13066140 | 4.24E-14 |  |  |  |  | 7.27E-09 |  |  |
|  | rs13088093 | 8.00E-10 |  |  |  |  |  |  |  |
|  | rs13109404 | 2.63E-18 |  |  | 2.46E-09 | 1.91E-07 |  |  | 1.04E-07 |
|  | rs1369159 | 1.65E-07 |  |  |  |  |  |  |  |
|  | rs138226610 |  |  |  |  |  |  | 6.28E-06 |  |
|  | rs1547026 | 1.10E-09 |  |  |  |  |  |  |  |
|  | rs174560 |  | 2.38E-06 |  |  |  |  |  |  |
|  | rs2048384 | 7.01E-06 |  |  |  |  | 1.31E-06 |  |  |
|  | rs2079070 |  |  |  |  | 4.84E-14 |  |  |  |
|  | rs2192528 | 6.86E-09 |  |  |  |  |  |  |  |
|  | rs2279681 | 1.00E-07 |  |  | 1.72E-07 |  |  |  |  |
|  | rs2717076 |  |  |  |  |  |  |  | 1.91E-06 |
|  | rs3027234 |  |  |  |  |  |  | 5.14E-07 |  |
|  | rs34556183 | 1.88E-07 |  |  | 1.28E-12 |  |  |  |  |
|  | rs34731055 | 2.12E-13 |  |  | 9.06E-06 | 4.38E-10 | 2.18E-06 |  | 1.76E-06 |
|  | rs3809162 | 2.72E-06 |  |  |  |  |  |  | 1.90E-16 |
|  | rs401966 |  |  |  |  | 9.85E-06 |  |  |  |
|  | rs41845 | 3.71E-07 |  |  |  |  |  |  |  |
|  | rs4364707 |  |  |  |  |  |  |  | 7.86E-07 |
|  | rs4428487 | 4.68E-09 |  |  |  |  |  |  |  |
|  | rs4592416 | 4.18E-10 |  | 8.10E-06 | 2.22E-07 | 6.93E-06 |  |  |  |
|  | rs4790581 |  |  |  | 3.17E-06 |  |  |  |  |
|  | rs4794053 |  | 1.35E-06 |  |  |  |  |  |  |
|  | rs4807177 | 5.93E-06 |  |  |  |  |  |  |  |
|  | rs4841498 | 4.07E-14 | 8.20E-06 |  | 4.39E-09 |  |  |  |  |
|  | rs4953152 |  |  |  |  |  |  | 9.85E-07 | 4.96E-07 |
|  | rs61796569 | 9.86E-06 |  |  |  |  |  |  |  |
|  | rs6575005 |  |  |  |  |  | 4.80E-06 |  |  |
|  | rs72771082 | 3.52E-07 |  |  |  |  |  |  | 2.06E-08 |
|  | rs73219758 | 4.33E-08 |  |  |  |  |  |  |  |
|  | rs73415247 |  |  |  | 8.46E-09 |  |  |  |  |
|  | rs74824115 | 1.26E-09 |  |  |  |  |  |  |  |
|  | rs780397 |  |  |  | 9.47E-06 |  |  | 8.94E-07 |  |
|  | rs7806045 |  |  |  |  | 9.06E-06 |  |  | 5.98E-06 |
|  | rs7842473 |  |  |  | 1.18E-08 |  |  |  |  |
|  | rs78677597 |  |  |  | 1.24E-08 |  |  |  |  |
|  | rs9552469 |  |  |  | 7.60E-06 |  |  |  |  |
|  | rs9915731 |  |  |  | 4.14E-07 |  |  |  |  |
|  | rs9937053 | 7.47E-58 | 2.72E-08 | 3.50E-24 | 3.04E-06 |  |  |  | 3.73E-09 |
| Short sleep (41) | rs10027044 | 2.63E-06 |  |  |  |  | 5.91E-07 |  |  |
|  | rs1008078 | 5.13E-08 |  |  |  | 1.34E-06 | 7.88E-11 | 4.18E-06 |  |
|  | rs1064939 | 4.93E-11 |  |  |  |  |  |  |  |
|  | rs1168948 |  |  |  |  |  | 9.79E-06 |  |  |
|  | rs11763750 | 5.31E-14 |  |  | 2.48E-06 | 4.31E-10 | 9.16E-07 |  | 4.61E-07 |
|  | rs1229762 |  |  |  |  | 7.92E-11 |  |  |  |
|  | rs12409424 |  |  |  |  |  |  |  | 4.74E-08 |
|  | rs12904938 | 1.02E-06 |  | 6.40E-10 | 1.64E-07 |  |  |  |  |
|  | rs13107325 | 2.80E-35 | 1.00E-15 |  | 1.65E-19 | 3.73E-09 |  |  | 7.88E-14 |
|  | rs1607227 | 4.37E-07 |  |  |  |  |  |  |  |
|  | rs17550821 | 8.23E-09 |  |  |  |  |  |  |  |
|  | rs1909204 | 8.21E-08 |  |  |  |  |  |  |  |
|  | rs2014830 | 2.77E-23 | 1.46E-07 |  | 5.26E-06 | 2.08E-06 |  |  | 4.36E-06 |
|  | rs2028263 | 6.50E-07 |  |  |  |  |  |  |  |
|  | rs2186122 | 1.20E-07 |  |  |  |  |  | 1.45E-06 |  |
|  | rs2517827 | 2.89E-09 |  |  |  |  |  | 3.62E-06 |  |
|  | rs2764264 | 3.11E-08 |  |  |  | 2.24E-06 |  |  |  |
|  | rs2820313 | 1.37E-27 |  |  | 6.06E-10 | 2.93E-07 |  |  |  |
|  | rs28409139 |  |  |  |  |  |  | 4.23E-07 |  |
|  | rs2863957 |  |  |  | 9.46E-06 |  |  |  |  |
|  | rs34786000 |  |  |  |  |  | 9.89E-06 |  |  |
|  | rs4014158 |  |  |  |  |  | 2.28E-10 |  |  |
|  | rs4894464 |  |  |  |  |  | 2.69E-06 |  |  |
|  | rs565629 |  |  |  | 9.04E-06 |  |  |  |  |
|  | rs613872 | 5.90E-08 |  |  |  | 9.12E-08 |  |  |  |
|  | rs675974 |  |  |  |  |  | 4.96E-08 |  |  |
|  | rs6774226 | 1.71E-09 |  |  |  |  |  |  |  |
|  | rs689143 |  |  |  | 8.09E-06 |  |  |  |  |
|  | rs7016567 | 6.21E-07 |  |  |  |  |  |  |  |
|  | rs7031064 | 2.25E-06 |  |  | 8.48E-08 |  |  |  |  |
|  | rs7132277 |  |  |  | 1.84E-11 | 1.23E-06 | 3.61E-14 |  |  |
|  | rs72979904 |  |  |  |  |  |  | 3.49E-06 |  |
|  | rs7299164 |  |  |  |  |  | 1.76E-07 |  |  |
|  | rs7350906 |  | 2.31E-08 |  |  |  |  |  |  |
|  | rs77883185 | 6.90E-09 |  |  |  |  |  |  |  |
|  | rs7842473 |  |  |  | 1.18E-08 |  |  |  |  |
|  | rs7895364 |  |  |  | 1.71E-08 |  |  |  |  |
|  | rs7922812 | 9.62E-09 |  |  |  |  |  |  |  |
|  | rs79269403 |  |  |  |  | 5.52E-06 |  |  |  |
|  | rs7939345 |  |  |  | 1.08E-07 |  |  |  |  |
|  | rs9649581 |  |  |  |  | 8.35E-06 |  |  | 1.22E-06 |
| Long sleep (23) | rs10241415 |  |  |  |  | 7.13E-06 |  |  |  |
|  | rs10939790 | 2.96E-09 |  |  |  |  |  |  |  |
|  | rs11634552 |  |  |  | 2.68E-06 |  |  |  |  |
|  | rs12499001 |  |  |  |  |  | 8.38E-07 |  |  |
|  | rs12524441 |  | 4.73E-06 |  |  |  |  |  |  |
|  | rs17817288 | 5.18E-43 | 3.92E-06 | 2.20E-21 | 7.59E-06 |  |  |  | 2.80E-06 |
|  | rs1947083 |  |  |  | 6.92E-07 |  |  |  |  |
|  | rs2251324 | 5.89E-06 |  |  |  |  |  |  |  |
|  | rs2393984 | 9.25E-10 |  |  | 1.14E-12 |  | 2.27E-08 |  | 4.00E-06 |
|  | rs2958492 |  |  |  |  | 7.66E-06 |  |  |  |
|  | rs330088 | 1.62E-14 |  |  | 3.80E-10 |  |  |  |  |
|  | rs34598277 | 2.97E-06 |  |  |  |  |  |  |  |
|  | rs4711989 | 1.69E-08 |  |  |  |  |  |  |  |
|  | rs571513 | 2.59E-12 | 4.45E-06 |  |  |  |  |  |  |
|  | rs62280752 |  |  |  |  |  |  | 1.47E-06 |  |
|  | rs6495126 |  |  |  | 2.45E-10 |  |  |  |  |
|  | rs6557516 |  |  |  |  | 4.59E-07 |  |  |  |
|  | rs6951574 |  |  |  |  |  |  | 1.71E-07 |  |
|  | rs7005575 |  |  |  |  |  | 2.82E-08 |  | 5.81E-08 |
|  | rs7534398 |  |  |  |  |  |  |  | 8.50E-06 |
|  | rs7581162 |  |  |  |  |  | 2.33E-10 | 1.18E-06 |  |
|  | rs766406 |  |  |  |  |  | 1.89E-08 | 1.40E-06 |  |
|  | rs77092939 |  |  |  |  |  | 6.24E-06 |  |  |
| Daytime napping (21) | rs1001817 | 1.60E-13 |  |  |  |  |  |  | 1.14E-06 |
|  | rs10257273 | 3.35E-12 |  |  |  | 7.74E-06 |  | 6.48E-06 |  |
|  | rs12140153 | 2.25E-14 |  | 7.83E-06 |  |  |  |  |  |
|  | rs17265513 |  |  | 8.64E-08 | 4.66E-06 |  |  |  |  |
|  | rs174541 |  | 2.56E-09 | 9.61E-21 |  |  |  |  |  |
|  | rs1931175 | 2.24E-11 |  |  |  |  |  |  |  |
|  | rs2143792 |  |  |  | 2.27E-06 |  |  |  |  |
|  | rs224111 |  |  |  | 8.43E-09 |  |  |  |  |
|  | rs2250377 | 4.74E-27 |  |  | 1.86E-10 |  |  |  |  |
|  | rs2431108 | 5.44E-06 |  |  |  |  | 4.56E-06 |  |  |
|  | rs2786547 | 2.81E-08 |  |  |  |  |  |  |  |
|  | rs35039375 | 2.49E-07 |  |  |  |  | 4.45E-07 |  | 1.14E-06 |
|  | rs378421 | 6.75E-27 |  |  |  | 2.32E-13 |  |  | 2.05E-19 |
|  | rs3935190 | 5.15E-06 |  |  |  |  |  |  |  |
|  | rs614987 | 8.89E-07 |  |  |  |  |  |  |  |
|  | rs62189006 |  |  |  | 2.59E-06 |  |  |  |  |
|  | rs6452787 |  |  |  |  |  |  | 6.53E-06 | 1.05E-08 |
|  | rs75022160 |  |  |  | 1.32E-06 |  |  |  |  |
|  | rs76824303 | 2.29E-08 |  |  |  |  |  |  |  |
|  | rs908442 | 7.37E-06 |  |  |  |  |  |  |  |
|  | rs9965170 | 6.90E-07 |  |  |  | 1.22E-06 | 2.38E-08 |  |  |
| Daytime sleepiness (38) | rs11123962 | 4.40E-07 |  |  |  |  |  | 4.04E-10 |  |
|  | rs12140153 | 2.25E-14 |  | 7.83E-06 |  |  |  |  |  |
|  | rs12554512 |  |  |  |  | 8.28E-07 | 1.94E-22 |  |  |
|  | rs12897149 |  |  |  |  |  |  |  | 1.41E-06 |
|  | rs13010456 | 4.40E-06 |  |  |  |  |  |  |  |
|  | rs13135092 | 2.60E-30 | 3.00E-13 |  | 1.19E-13 | 6.44E-09 |  |  | 1.48E-14 |
|  | rs13149643 | 1.94E-07 |  |  |  |  |  |  |  |
|  | rs1566362 |  |  |  |  |  |  | 7.74E-07 |  |
|  | rs1858513 |  |  |  |  |  | 4.77E-06 |  |  |
|  | rs1888489 | 9.55E-06 |  |  |  |  |  |  |  |
|  | rs1937444 | 6.35E-06 |  |  |  |  |  |  |  |
|  | rs2048522 |  |  |  |  |  | 4.73E-07 |  |  |
|  | rs2160515 |  |  |  |  |  |  | 4.44E-07 |  |
|  | rs224109 |  |  |  | 1.36E-09 |  |  |  |  |
|  | rs2390669 | 2.00E-10 |  |  |  |  |  |  |  |
|  | rs2472297 | 9.00E-08 |  |  | 6.75E-06 |  |  |  |  |
|  | rs2652457 |  |  |  |  |  |  | 5.27E-06 |  |
|  | rs2733486 |  |  |  | 2.28E-06 |  |  |  |  |
|  | rs2787120 | 6.67E-09 |  |  |  |  |  |  |  |
|  | rs28768443 |  |  |  |  | 8.25E-06 |  |  |  |
|  | rs3180887 |  |  |  |  |  |  |  | 2.09E-06 |
|  | rs325501 | 4.86E-07 |  |  |  |  |  |  |  |
|  | rs4432411 |  |  |  | 5.87E-06 |  |  | 3.36E-06 |  |
|  | rs4665972 |  | 1.86E-30 | 3.90E-09 |  |  |  |  | 1.29E-36 |
|  | rs506751 |  |  |  |  |  | 7.54E-06 |  |  |
|  | rs57746981 | 1.55E-10 |  |  | 2.71E-06 |  |  |  |  |
|  | rs62055936 |  |  |  | 9.48E-09 | 8.55E-08 | 3.27E-07 |  | 2.95E-10 |
|  | rs62505473 | 2.85E-06 |  |  |  |  |  |  | 5.36E-06 |
|  | rs641498 | 8.51E-07 |  |  |  |  |  |  |  |
|  | rs6741951 | 2.92E-18 |  |  |  |  |  |  | 4.42E-06 |
|  | rs7605591 |  |  |  | 8.92E-17 |  |  |  |  |
|  | rs7607363 | 7.99E-10 |  |  |  |  |  |  |  |
|  | rs762995 |  |  |  |  |  |  | 1.27E-06 |  |
|  | rs8059398 |  |  |  | 4.57E-06 |  |  |  |  |
|  | rs843372 | 5.38E-08 |  |  |  |  |  |  |  |
|  | rs960986 | 7.23E-14 |  |  |  |  | 2.78E-06 | 1.47E-10 |  |
|  | rs9712275 | 1.92E-08 |  |  |  |  |  |  |  |
|  | rs9947450 | 9.08E-06 |  |  |  |  |  |  |  |
| Chronotype (35) | rs1064213 | 1.70E-11 |  |  |  |  |  |  |  |
|  | rs10742179 | 9.49E-22 |  |  |  |  |  | 1.53E-07 |  |
|  | rs11588913 | 1.56E-06 |  |  |  |  |  |  |  |
|  | rs11712056 | 2.36E-20 |  |  |  | 8.96E-08 | 7.53E-24 |  | 9.13E-06 |
|  | rs11748798 |  |  |  |  |  |  | 8.58E-07 |  |
|  | rs11786306 | 1.54E-06 |  |  |  |  |  |  |  |
|  | rs12055234 | 2.61E-06 |  |  |  |  |  |  |  |
|  | rs12140153 | 2.25E-14 |  | 7.83E-06 |  |  |  |  |  |
|  | rs139911 | 6.63E-09 |  |  |  |  |  |  |  |
|  | rs1421085 | 4.00E-15 | 2.33E-08 | 1.80E-24 | 1.82E-15 |  |  |  | 1.07E-09 |
|  | rs17161045 | 1.95E-06 |  |  |  |  | 3.62E-06 | 2.79E-06 |  |
|  | rs197273 |  |  |  |  |  |  |  | 7.00E-08 |
|  | rs28380327 |  |  |  | 2.65E-06 | 6.07E-06 | 1.21E-12 |  |  |
|  | rs28647842 |  |  |  |  |  |  | 4.06E-06 |  |
|  | rs286805 | 9.71E-06 |  |  |  |  |  |  |  |
|  | rs2971970 |  |  |  |  | 2.29E-06 | 2.51E-08 |  |  |
|  | rs308521 | 3.12E-06 |  |  | 4.13E-06 |  |  |  |  |
|  | rs3168135 |  | 3.80E-07 |  | 3.65E-06 |  |  |  |  |
|  | rs359250 |  |  |  |  |  |  | 3.68E-07 |  |
|  | rs3808477 | 3.22E-12 | 7.36E-07 |  |  |  |  |  |  |
|  | rs4237555 |  |  | 1.30E-06 |  |  |  |  |  |
|  | rs4241964 | 7.66E-13 |  |  |  |  |  |  |  |
|  | rs4729854 |  |  | 4.59E-07 |  |  |  |  |  |
|  | rs521977 |  | 5.94E-11 | 2.76E-12 | 7.06E-08 |  |  |  |  |
|  | rs56947091 |  |  |  |  | 2.90E-06 | 2.51E-10 |  |  |
|  | rs62405438 | 1.85E-34 |  | 1.08E-07 |  |  |  |  |  |
|  | rs6967481 | 9.85E-06 |  |  |  |  |  |  |  |
|  | rs72632979 | 5.45E-07 |  |  |  |  |  |  |  |
|  | rs72720396 |  |  |  |  |  | 7.76E-06 |  | 1.13E-12 |
|  | rs7959983 |  |  |  | 6.37E-06 |  |  |  |  |
|  | rs812925 |  |  |  | 3.22E-06 |  |  |  |  |
|  | rs9395520 | 1.52E-09 |  |  |  | 5.57E-06 |  |  |  |
|  | rs957501 |  |  |  |  |  |  | 2.08E-06 |  |
|  | rs9636202 | 2.06E-11 |  |  | 3.60E-07 |  |  |  |  |
|  | rs9932577 | 2.54E-10 |  |  |  |  |  |  |  |
| Morning person (25) | rs10501087 | 3.26E-14 |  |  |  |  |  | 7.22E-09 |  |
|  | rs11229543 |  | 4.28E-07 |  | 3.83E-06 |  |  |  |  |
|  | rs11588913 | 1.56E-06 |  |  |  |  |  |  |  |
|  | rs11645898 |  |  |  |  |  |  |  | 6.18E-06 |
|  | rs11712056 | 2.36E-20 |  |  |  | 8.96E-08 | 7.53E-24 |  | 9.13E-06 |
|  | rs11786306 | 1.54E-06 |  |  |  |  |  |  |  |
|  | rs12055234 | 2.61E-06 |  |  |  |  |  |  |  |
|  | rs12140153 | 2.25E-14 |  | 7.83E-06 |  |  |  |  |  |
|  | rs12669911 | 1.00E-06 |  |  |  |  | 2.72E-06 | 7.69E-06 |  |
|  | rs12682033 | 2.20E-09 |  |  |  |  |  |  |  |
|  | rs139911 | 6.63E-09 |  |  |  |  |  |  |  |
|  | rs1421085 | 4.00E-15 | 2.33E-08 | 1.80E-24 | 1.82E-15 |  |  |  | 1.07E-09 |
|  | rs28380327 |  |  |  | 2.65E-06 | 6.07E-06 | 1.21E-12 |  |  |
|  | rs308521 | 3.12E-06 |  |  | 4.13E-06 |  |  |  |  |
|  | rs3767240 |  |  |  | 9.12E-06 |  |  |  |  |
|  | rs4241964 | 7.66E-13 |  |  |  |  |  |  |  |
|  | rs4729854 |  |  | 4.59E-07 |  |  |  |  |  |
|  | rs6967481 | 9.85E-06 |  |  |  |  |  |  |  |
|  | rs72632979 | 5.45E-07 |  |  |  |  |  |  |  |
|  | rs72720396 |  |  |  |  |  | 7.76E-06 |  | 1.13E-12 |
|  | rs778147 |  |  |  | 4.24E-07 |  |  |  |  |
|  | rs7959983 |  |  |  | 6.37E-06 |  |  |  |  |
|  | rs9369915 | 2.83E-09 |  |  |  | 4.90E-06 |  |  |  |
|  | rs957501 |  |  |  |  |  |  | 2.08E-06 |  |
|  | rs9636202 | 2.06E-11 |  |  | 3.60E-07 |  |  |  |  |
| Snoring (47) | rs10150432 | 9.86E-06 |  |  |  | 6.98E-06 |  |  |  |
|  | rs10505911 | 6.47E-06 |  |  |  |  |  |  |  |
|  | rs10740047 |  |  |  |  |  | 1.26E-06 |  |  |
|  | rs10760677 |  |  |  |  |  |  | 7.79E-06 |  |
|  | rs11041980 | 4.56E-07 |  |  |  |  |  |  |  |
|  | rs111836343 | 4.24E-08 |  |  |  |  |  |  |  |
|  | rs115950813 | 4.72E-06 |  |  |  |  |  |  |  |
|  | rs11616501 | 8.08E-09 |  |  |  |  |  |  |  |
|  | rs11639856 | 1.45E-07 |  |  | 7.95E-07 |  |  |  |  |
|  | rs11690369 | 1.63E-08 |  |  |  |  |  |  |  |
|  | rs12449843 |  |  |  | 1.44E-07 |  |  |  |  |
|  | rs12724444 |  |  |  |  |  | 8.05E-11 |  |  |
|  | rs12765002 | 1.22E-08 |  |  | 2.72E-07 |  |  | 2.92E-09 |  |
|  | rs140106922 | 2.86E-08 |  |  |  |  |  |  |  |
|  | rs147730268 | 7.38E-17 |  |  |  |  |  |  |  |
|  | rs1582931 | 8.97E-10 |  |  | 2.79E-12 |  |  |  |  |
|  | rs16952896 | 8.13E-08 |  |  |  |  |  |  |  |
|  | rs17804073 | 3.16E-09 |  |  |  |  |  | 3.08E-06 |  |
|  | rs1915792 | 3.19E-09 |  |  |  |  |  | 8.55E-06 |  |
|  | rs2049045 | 5.66E-14 |  |  |  |  |  | 2.04E-09 |  |
|  | rs2239842 |  |  | 1.35E-06 |  |  |  |  |  |
|  | rs2307111 | 1.13E-07 | 7.34E-28 | 6.23E-08 |  |  |  |  |  |
|  | rs2683618 |  |  |  | 5.46E-06 |  |  |  |  |
|  | rs34811474 | 4.40E-20 |  |  | 2.59E-08 |  |  |  |  |
|  | rs34821122 | 5.10E-08 |  |  |  |  |  |  |  |
|  | rs34838 | 3.10E-23 |  |  |  | 4.83E-12 |  |  | 1.69E-18 |
|  | rs34929358 | 1.31E-07 |  |  |  |  |  |  |  |
|  | rs4017425 | 4.46E-09 |  |  |  |  |  |  |  |
|  | rs4647709 |  | 7.93E-08 |  |  |  |  |  |  |
|  | rs4766566 |  |  |  | 4.19E-14 |  |  |  |  |
|  | rs4815915 | 1.28E-16 |  |  |  |  |  |  |  |
|  | rs4818008 | 9.53E-09 |  |  |  |  |  |  |  |
|  | rs56396135 |  |  |  |  |  |  | 5.21E-06 |  |
|  | rs57222984 |  |  |  | 3.93E-07 | 4.21E-06 | 1.37E-06 |  | 5.27E-10 |
|  | rs59208569 | 1.71E-11 |  |  | 4.08E-06 |  | 8.21E-06 |  |  |
|  | rs61903695 | 2.78E-09 |  |  |  |  |  |  |  |
|  | rs62158213 |  |  |  | 7.29E-06 |  |  |  |  |
|  | rs6475736 |  |  |  |  |  | 3.08E-11 |  |  |
|  | rs6491430 | 8.93E-06 |  |  |  |  |  |  |  |
|  | rs6722241 | 1.06E-13 |  |  |  | 2.00E-06 | 3.10E-13 |  |  |
|  | rs73024804 | 3.12E-10 |  |  |  |  |  |  |  |
|  | rs732172 | 1.56E-08 |  |  |  |  |  |  |  |
|  | rs7514002 | 2.32E-09 |  |  |  |  |  |  |  |
|  | rs7924036 | 4.16E-10 |  |  | 1.28E-11 |  | 4.48E-09 |  | 3.67E-07 |
|  | rs8047587 | ######## |  | 1.40E-22 | 6.42E-13 |  |  |  | 3.61E-09 |
|  | rs8133137 | 2.12E-07 |  |  |  |  |  |  |  |
|  | rs8176746 |  | 2.29E-08 |  |  |  |  |  |  |
| Apnea–hypopnea index (1) | rs17476364 |  | 4.92E-06 |  |  |  |  |  |  |
| MDD (16) | rs10039321 |  |  |  |  |  |  | 9.75E-09 |  |
|  | rs11121207 |  |  |  | 3.79E-06 |  |  |  |  |
|  | rs1463728 | 1.01E-06 |  |  |  |  | 1.65E-07 |  |  |
|  | rs1548461 |  |  |  |  |  | 1.35E-07 | 1.32E-07 |  |
|  | rs17543112 |  |  |  |  | 1.75E-06 |  |  |  |
|  | rs2056476 |  |  |  |  |  |  | 7.91E-09 |  |
|  | rs2509805 |  |  |  | 4.10E-07 | 2.05E-09 |  |  | 2.17E-06 |
|  | rs372519 | 2.42E-13 |  |  |  |  |  |  |  |
|  | rs4776768 | 4.61E-06 |  |  |  |  |  |  |  |
|  | rs5011520 | 1.74E-07 |  |  | 2.22E-14 |  |  |  |  |
|  | rs61533748 |  |  |  |  |  |  | 2.96E-06 |  |
|  | rs6832890 |  |  |  |  |  |  |  | 4.36E-07 |
|  | rs6905391 | 5.08E-07 |  | 9.59E-06 | 1.50E-08 |  |  |  |  |
|  | rs726857 |  |  |  | 1.51E-14 |  |  |  |  |
|  | rs7531118 | 6.38E-14 |  |  | 7.86E-07 |  | 1.84E-06 |  |  |
|  | rs76025409 |  |  |  |  |  | 5.14E-06 |  |  |

*Exposure includes insomnia, sleep duration, long sleep, daytime napping, daytime sleepiness, chronotype, morning person, snoring, and apnea–hypopnea index. Mediator means MDD.

†The main indicators of obesity included BMI and WHR; blood lipids included LDL cholesterol, HDL cholesterol, and triglycerides; blood glucose included fasting glucose/insulin and type 2 diabetes; blood pressure included SBP, DBP, and hypertension; physical activity included number of days or week and types of physical activity; education included level and years of education; smoking included past and current tobacco smoking; drinking included alcohol consumption and intake frequency. The *P*-value association with confounders can be obtained using the PhenoScanner tool at http://www.phenoscanner.medschl.cam.ac.uk/.

## Table S3. Details of removed SNPs (missing, ambiguous palindromic, and outlier).

| Exposure/  Mediator* | Mediator/  Outcome† | Remove SNPs missing in the outcome | Remove ambiguous palindromic SNPs | Remove outlier SNPs |
| --- | --- | --- | --- | --- |
| Insomnia | MDD | NA | rs10978481; rs1125988; rs12403670; rs1731951; rs1870736; rs2147141; rs4767643; rs504403; rs582122; rs8074498 | rs12531550; rs79469956; rs117872294; rs575346808 |
|  | CAD | rs114345591; rs142626045; rs145638045; rs149025697; rs575346808; rs79575047; rs8082416; rs9770953 | rs10978481; rs1125988; rs12403670; rs1731951; rs2147141; rs4767643; rs504403; rs582122; rs8074498 | NA |
|  | MI | rs114345591; rs114871329; rs142613636; rs142626045; rs145638045; rs149025697; rs575346808; rs79575047; rs8082416; rs9770953 | rs10978481; rs1125988; rs12403670; rs1731951; rs2147141; rs4767643; rs504403; rs582122; rs8074498 | NA |
|  | HF | rs117872294; rs142626045; rs145524905; rs145638045; rs151300568; rs575346808; rs78190272; rs79575047; rs8082416; rs9770953 | rs10978481; rs1125988; rs12403670; rs1731951; rs1870736; rs2147141; rs4767643; rs504403; rs582122; rs8074498 | NA |
|  | AF | NA | rs10978481; rs1125988; rs12403670; rs1731951; rs1870736; rs2147141; rs4767643; rs504403; rs582122; rs8074498 | NA |
|  | Stroke | rs142626045; rs145638045; rs575346808; rs78190272; rs79575047; rs9770953 | rs10978481; rs1125988; rs12403670; rs1731951; rs1870736; rs2147141; rs4767643; rs504403; rs582122; rs8074498 | NA |
| Sleep duration | MDD | rs11853131 | rs11659881; rs17732997; rs1809498; rs269054; rs35236861; rs55842233; rs6425618; rs6533695; rs7178161; rs7943526; rs8074498 | rs10273733; rs10973207; rs11738968 |
|  | CAD | rs111304747; rs112398654; rs143222942; rs186871508; rs28754455; rs8072993 | rs11659881; rs17732997; rs1809498; rs269054; rs35236861; rs55842233; rs6425618; rs6533695; rs7178161; rs7943526; rs8074498 | rs4473312 |
|  | MI | rs111304747; rs112398654; rs143222942; rs186871508; rs28754455; rs8072993 | rs11659881; rs17732997; rs1809498; rs269054; rs35236861; rs55842233; rs6425618; rs6533695; rs7178161; rs7943526; rs8074498 | NA |
|  | HF | rs111304747; rs112398654; rs147114641; rs186871508; rs28754455; rs4376202; rs74800771; rs78842483; rs8072993 | rs11659881; rs17732997; rs1809498; rs269054; rs35236861; rs55842233; rs6425618; rs6533695; rs7178161; rs7460584; rs7943526; rs8074498 | NA |
|  | AF | rs147114641 | rs11659881; rs12336359; rs17732997; rs1809498; rs269054; rs35236861; rs55842233; rs6425618; rs6533695; rs7178161; rs7460584; rs7943526; rs8074498 | rs10273733; rs10820727; rs11190970; rs12366583; rs2696429; rs28656154 |
|  | Stroke | rs111304747; rs112398654; rs186871508; rs28754455; rs72672112; rs78842483; rs8072993 | rs11659881; rs17732997; rs1809498; rs269054; rs35236861; rs55842233; rs6425618; rs6533695; rs7178161; rs7460584; rs7943526; rs8074498 | rs1424324 |
| Short sleep | MDD | NA | rs11043328; rs1751290; rs60483752; rs7134646; rs7625629; rs8074498; rs9367621; rs963474 | rs12963463; rs6741228 |
|  | CAD | rs11148392; rs117579512; rs12047989; rs138457055; rs147657557 | rs11043328; rs11698090; rs1751290; rs2079987; rs59884956; rs60483752; rs7134646; rs7625629; rs8074498; rs9367621; rs963474 | NA |
|  | MI | rs11148392; rs117579512; rs12047989; rs138457055; rs147657557; rs71437291 | rs11043328; rs11698090; rs1751290; rs2079987; rs4392510; rs59884956; rs60483752; rs7134646; rs7625629; rs8074498; rs9367621; rs963474 | NA |
|  | HF | rs11148392; rs117579512; rs12047989; rs138630664; rs143681554; rs147657557; rs1818814; rs76429071; rs78702570 | rs11043328; rs1751290; rs60483752; rs7134646; rs7625629; rs8074498; rs9367621; rs963474 | NA |
|  | AF | NA | rs11043328; rs11698090; rs1751290; rs2056914; rs2079987; rs4392510; rs59884956; rs60483752; rs7134646; rs7625629; rs8074498; rs9367621; rs963474 | rs10820727; rs9745431 |
|  | Stroke | rs11148392; rs117579512; rs12047989; rs138630664; rs76429071 | rs11043328; rs11698090; rs1751290; rs2079987; rs60483752; rs7134646; rs7625629; rs8074498; rs9367621; rs963474 | NA |
| Long sleep | MDD | rs111638191; rs572857764; rs77875625 | rs40430 | NA |
|  | CAD | rs111638191; rs117843320; rs146467757; rs572857764; rs7450155; rs76141296; rs77875625; rs78686210 | rs40430 | NA |
|  | MI | rs111638191; rs114567224; rs117843320; rs146467757; rs572857764; rs73169613; rs7450155; rs76141296; rs77875625; rs78686210 | rs40430 | NA |
|  | HF | rs111638191; rs117843320; rs138038426; rs146467757; rs147114641; rs17109989; rs572857764; rs62073915; rs72701154; rs7450155; rs76141296; rs77698412; rs77875625 | rs40430 | rs35794765 |
|  | AF | rs111638191; rs146467757; rs147114641; rs572857764; rs62073915 | rs40430 | rs4508677; rs76014553 |
|  | Stroke | rs111638191; rs146467757; rs17109989; rs572857764; rs7450155; rs76141296; rs77698412; rs77875625 | rs40430 | rs4858192 |
| Daytime napping | MDD | NA | rs2417268; rs285815 | rs35570980; rs3799380; rs910187 |
|  | CAD | NA | rs2417268; rs285815 | NA |
|  | MI | NA | rs2417268; rs285815 | NA |
|  | HF | NA | rs2417268; rs285815 | NA |
|  | AF | rs4511908 | rs2417268; rs285815 | rs385199 |
|  | Stroke | NA | rs2417268; rs285815 | NA |
| Daytime sleepiness | MDD | rs3009841; rs3062868 | rs10822589; rs12706050; rs151285; rs4486037 | rs35284403 |
|  | CAD | rs138064993; rs139930100; rs148606436; rs1516485; rs3009841; rs3062868; rs6940935; rs75510037 | rs10822589; rs12706050; rs151285; rs4486037; rs4765939; rs4917266 | NA |
|  | MI | rs138064993; rs139930100; rs148588565; rs148606436; rs1516485; rs3009841; rs3062868; rs6940935; rs75510037 | rs10822589; rs12706050; rs151285; rs4486037; rs4917266 | NA |
|  | HF | rs138064993; rs139476608; rs139930100; rs140622509; rs147114641; rs148606436; rs149004796; rs149990204; rs1516485; rs186751163; rs3009841; rs3062868; rs6940935; rs75510037; rs79635214 | rs10822589; rs12706050; rs151285; rs4486037 | NA |
|  | AF | rs139476608; rs147114641; rs3009841 | rs10822589; rs12706050; rs151285; rs4486037; rs4765939; rs4917266 | rs4743034 |
|  | Stroke | rs138064993; rs139476608; rs139930100; rs149004796; rs1516485; rs3009841; rs3062868; rs6940935; rs75510037 | rs10822589; rs12706050; rs151285; rs4486037 | NA |
| Chronotype | MDD | NA | rs9962650 | NA |
|  | CAD | rs28458909 | rs9962650 | NA |
|  | MI | rs28458909 | rs9962650 | NA |
|  | HF | NA | rs9962650 | NA |
|  | AF | NA | rs9962650 | NA |
|  | Stroke | NA | rs9962650 | rs17575798 |
| Morning person | MDD | NA | rs12498561; rs512647; rs9962650 | NA |
|  | CAD | rs28458909 | rs12498561; rs512647; rs9962650 | NA |
|  | MI | rs28458909 | rs12498561; rs512647; rs9962650 | NA |
|  | HF | NA | rs12498561; rs512647; rs9962650 | NA |
|  | AF | NA | rs12498561; rs512647; rs9962650 | rs11679484 |
|  | Stroke | NA | rs12498561; rs512647; rs9962650 | rs17575798 |
| Snoring | MDD | NA | rs10475978 rs180107; rs4320625 | rs11746047; rs60465570 |
|  | CAD | rs76605772 | rs10475978; rs12185006; rs180107; rs4320625 | NA |
|  | MI | rs116292849; rs2072104; rs76605772 | rs10475978; rs12185006; rs13008952; rs180107; rs4320625; rs9583546 | rs1459331 |
|  | HF | rs112552581; rs144189373; rs74356841 | rs10475978; rs4320625 | rs1016013 |
|  | AF | NA | rs10475978; rs12185006; rs13008952; rs180107; rs4320625; rs9583546 | rs1016013; rs11103374; rs1480036; rs1641511; rs2052220; rs34565348 |
|  | Stroke | NA | rs10475978; rs4320625 | NA |
| Apnea–hypopnea index | MDD | NA | rs1510230; rs579339; rs6918923 | NA |
|  | CAD | rs6450070 | rs1510230; rs6918923 | NA |
|  | MI | rs113081935; rs6450070 | rs1510230; rs6918923 | NA |
|  | HF | rs113081935; rs115163485; rs6450070 | rs1510230; rs579339; rs6918923 | NA |
|  | AF | rs6450070 | rs1510230; rs579339; rs6918923 | NA |
|  | Stroke | rs113081935; rs6450070 | rs1510230; rs579339; rs6918923 | NA |
| MDD | CAD | rs10411666; rs115670873; rs117141160; rs117473501; rs141858113; rs368352425; rs78338135 | rs10759934; rs1152589; rs1214608 | NA |
|  | MI | rs10411666; rs115670873; rs117141160; rs117473501; rs141858113; rs144926805; rs368352425; rs7558022; rs78338135 | rs10759934; rs1152589; rs1214608; rs890068 | NA |
|  | HF | rs115670873; rs117141160; rs117473501; rs139627276; rs141487324; rs141811146; rs141858113; rs144926805; rs184397922; rs368352425; rs75208528; rs78338135 | rs10759934; rs1152589; rs1214608 | NA |
|  | AF | rs141487324; rs184397922; rs368352425; rs4667368; rs7117176 | rs10759934; rs1152589; rs1214608; rs890068 | NA |
|  | Stroke | rs141858113; rs184397922; rs368352425; rs75208528 | rs10759934; rs1152589; rs1214608 | NA |

MDD, major depressive disorder; CAD, coronary artery disease; MI, myocardial infarction; HF, heart failure; AF, atrial fibrillation.

*Exposure includes insomnia, sleep duration, long sleep, daytime napping, daytime sleepiness, chronotype, morning person, snoring, and apnea–hypopnea index. Mediator means MDD.

†Mediator means MDD. Outcome includes CAD, MI, HF, AF, and Stroke.

## Table S4. The priori statistical power in the MR analysis of the causal effects.

| Exposure/  Mediator* | Mediator/  Outcome† | No. SNPs | Sample size | Cases | K‡ | F-statistic (mean) | Minimum detectable OR | |
| --- | --- | --- | --- | --- | --- | --- | --- | --- |
|  |  |  |  |  |  |  | OR <1 | OR >1 |
| Insomnia | MDD | 273 | 173,005 | 59,851 | 0.346 | 23.09 | 0.92 | 1.09 |
|  | CAD | 270 | 184,305 | 60,801 | 0.33 | 23.11 | 0.92 | 1.09 |
|  | MI | 268 | 171,875 | 43,676 | 0.254 | 23.11 | 0.91 | 1.10 |
|  | HF | 267 | 977,323 | 47,309 | 0.048 | 23.15 | 0.92 | 1.08 |
|  | AF | 277 | 588,190 | 65,446 | 0.111 | 23.13 | 0.93 | 1.07 |
|  | Stroke | 271 | 446,696 | 40,585 | 0.091 | 23.1 | 0.92 | 1.09 |
| Sleep duration | MDD | 320 | 173,005 | 59,851 | 0.346 | 23.87 | 0.90 | 1.11 |
|  | CAD | 317 | 184,305 | 60,801 | 0.33 | 23.99 | 0.90 | 1.11 |
|  | MI | 318 | 171,875 | 43,676 | 0.254 | 23.98 | 0.89 | 1.12 |
|  | HF | 314 | 977,323 | 47,309 | 0.048 | 23.96 | 0.91 | 1.10 |
|  | AF | 315 | 588,190 | 65,446 | 0.111 | 23.86 | 0.92 | 1.09 |
|  | Stroke | 315 | 446,696 | 40,585 | 0.091 | 23.97 | 0.90 | 1.11 |
| Short sleep | MDD | 234 | 173,005 | 59,851 | 0.346 | 21.26 | 0.88 | 1.14 |
|  | CAD | 228 | 184,305 | 60,801 | 0.33 | 21.41 | 0.88 | 1.13 |
|  | MI | 226 | 171,875 | 43,676 | 0.254 | 21.4 | 0.87 | 1.15 |
|  | HF | 227 | 977,323 | 47,309 | 0.048 | 21.46 | 0.89 | 1.12 |
|  | AF | 229 | 588,190 | 65,446 | 0.111 | 21.37 | 0.90 | 1.11 |
|  | Stroke | 229 | 446,696 | 40,585 | 0.091 | 21.42 | 0.88 | 1.14 |
| Long sleep | MDD | 147 | 173,005 | 59,851 | 0.346 | 20.74 | 0.86 | 1.16 |
|  | CAD | 142 | 184,305 | 60,801 | 0.33 | 20.65 | 0.87 | 1.16 |
|  | MI | 140 | 171,875 | 43,676 | 0.254 | 20.67 | 0.85 | 1.18 |
|  | HF | 136 | 977,323 | 47,309 | 0.048 | 20.3 | 0.87 | 1.15 |
|  | AF | 143 | 588,190 | 65,446 | 0.111 | 20.29 | 0.89 | 1.13 |
|  | Stroke | 141 | 446,696 | 40,585 | 0.091 | 20.68 | 0.86 | 1.16 |
| Daytime napping | MDD | 78 | 173,005 | 59,851 | 0.346 | 44.9 | 0.59 | 1.70 |
|  | CAD | 81 | 184,305 | 60,801 | 0.33 | 44.6 | 0.86 | 1.16 |
|  | MI | 81 | 171,875 | 43,676 | 0.254 | 44.6 | 0.85 | 1.18 |
|  | HF | 81 | 977,323 | 47,309 | 0.048 | 44.6 | 0.87 | 1.15 |
|  | AF | 79 | 588,190 | 65,446 | 0.111 | 42.64 | 0.88 | 1.14 |
|  | Stroke | 81 | 446,696 | 40,585 | 0.091 | 44.6 | 0.86 | 1.17 |
| Daytime sleepiness | MDD | 241 | 173,005 | 59,851 | 0.346 | 22.31 | 0.88 | 1.14 |
|  | CAD | 234 | 184,305 | 60,801 | 0.33 | 22.3 | 0.88 | 1.14 |
|  | MI | 234 | 171,875 | 43,676 | 0.254 | 22.37 | 0.67 | 1.50 |
|  | HF | 229 | 977,323 | 47,309 | 0.048 | 22.37 | 0.89 | 1.13 |
|  | AF | 238 | 588,190 | 65,446 | 0.111 | 22.17 | 0.90 | 1.11 |
|  | Stroke | 235 | 446,696 | 40,585 | 0.091 | 22.41 | 0.88 | 1.14 |
| Chronotype | MDD | 116 | 173,005 | 59,851 | 0.346 | 48.54 | 0.88 | 1.13 |
|  | CAD | 115 | 184,305 | 60,801 | 0.33 | 47.96 | 0.88 | 1.13 |
|  | MI | 115 | 171,875 | 43,676 | 0.254 | 47.96 | 0.87 | 1.15 |
|  | HF | 116 | 977,323 | 47,309 | 0.048 | 48.54 | 0.89 | 1.12 |
|  | AF | 116 | 588,190 | 65,446 | 0.111 | 48.54 | 0.90 | 1.11 |
|  | Stroke | 115 | 446,696 | 40,585 | 0.091 | 48.57 | 0.88 | 1.13 |
| Morning person | MDD | 94 | 173,005 | 59,851 | 0.346 | 46.55 | 0.87 | 1.14 |
|  | CAD | 93 | 184,305 | 60,801 | 0.33 | 45.98 | 0.88 | 1.14 |
|  | MI | 93 | 171,875 | 43,676 | 0.254 | 45.98 | 0.86 | 1.16 |
|  | HF | 94 | 977,323 | 47,309 | 0.048 | 46.55 | 0.89 | 1.13 |
|  | AF | 93 | 588,190 | 65,446 | 0.111 | 46.61 | 0.90 | 1.11 |
|  | Stroke | 93 | 446,696 | 40,585 | 0.091 | 46.53 | 0.87 | 1.14 |
| Snoring | MDD | 242 | 173,005 | 59,851 | 0.346 | 22.48 | 0.89 | 1.12 |
|  | CAD | 242 | 184,305 | 60,801 | 0.33 | 22.5 | 0.89 | 1.12 |
|  | MI | 237 | 171,875 | 43,676 | 0.254 | 22.54 | 0.88 | 1.13 |
|  | HF | 241 | 977,323 | 47,309 | 0.048 | 22.51 | 0.90 | 1.11 |
|  | AF | 235 | 588,190 | 65,446 | 0.111 | 22.39 | 0.91 | 1.10 |
|  | Stroke | 245 | 446,696 | 40,585 | 0.091 | 22.54 | 0.89 | 1.12 |
| Apnea–hypopnea index | MDD | 61 | 173,005 | 59,851 | 0.346 | 34.42 | 0.99 | 1.01 |
|  | CAD | 61 | 184,305 | 60,801 | 0.33 | 34.2 | 0.99 | 1.01 |
|  | MI | 60 | 171,875 | 43,676 | 0.254 | 32.32 | 0.99 | 1.01 |
|  | HF | 58 | 977,323 | 47,309 | 0.048 | 30.83 | 0.99 | 1.01 |
|  | AF | 60 | 588,190 | 65,446 | 0.111 | 34.26 | 0.99 | 1.01 |
|  | Stroke | 59 | 446,696 | 40,585 | 0.091 | 32.37 | 0.99 | 1.01 |
| MDD | CAD | 181 | 184,305 | 60,801 | 0.33 | 20.05 | 0.91 | 1.10 |
|  | MI | 178 | 171,875 | 43,676 | 0.254 | 20.05 | 0.99 | 1.01 |
|  | HF | 176 | 977,323 | 47,309 | 0.048 | 20.07 | 0.91 | 1.09 |
|  | AF | 182 | 588,190 | 65,446 | 0.111 | 20.1 | 0.92 | 1.08 |
|  | Stroke | 184 | 446,696 | 40,585 | 0.091 | 20.07 | 0.91 | 1.10 |

MDD, major depressive disorder; CAD, coronary artery disease; MI, myocardial infarction; HF, heart failure; AF, atrial fibrillation.

*Exposure includes insomnia, sleep duration, long sleep, daytime napping, daytime sleepiness, chronotype, morning person, snoring, and apnea–hypopnea index. Mediator means MDD.

†Mediator means MDD. Outcome includes CAD, MI, HF, AF, and Stroke.

‡K means proportion of cases in the study

## Table S5. The pleiotropy and heterogeneity for primary univariate MR analyses of sleep traits on CVDs.

| Exposure | Outcome | No. SNPs | MR-Egger intercept test | | Cochran’s Q-statistic test † | | |
| --- | --- | --- | --- | --- | --- | --- | --- |
|  |  |  | Intercept (95% CI) | *P*-value | Q-statistic | I^2^, %* | *P*-value |
| Insomnia | CAD | 270 | 0.002 (-0.003-0.007) | 5.07E-01 | 295.9 | 9.09 | 1.25E-01 |
|  | MI | 268 | 0.000 (-0.005-0.006) | 9.63E-01 | 283.74 | 5.9 | 2.30E-01 |
|  | HF | 267 | -0.004 (-0.009-0.000) | 5.30E-02 | 326.91 | 18.63 | 6.37E-03 |
|  | AF | 277 | 0.003 (-0.001-0.008) | 1.28E-01 | 383.11 | **27.96** | **2.04E-05** |
|  | Stroke | 271 | 0.001 (-0.005-0.007) | 7.20E-01 | 374.79 | **27.96** | **2.47E-05** |
| Sleep duration | CAD | 317 | 0.002 (-0.003-0.006) | 4.46E-01 | 347.03 | 8.94 | 8.09E-02 |
|  | MI | 318 | -0.002 (-0.006-0.003) | 5.25E-01 | 330.95 | 4.22 | 2.83E-01 |
|  | HF | 314 | -0.001 (-0.005-0.002) | 4.89E-01 | 328.61 | 4.75 | 2.61E-01 |
|  | AF | 315 | -0.002 (-0.006-0.002) | 2.59E-01 | 382.55 | 17.92 | 4.88E-03 |
|  | Stroke | 315 | 0.003 (-0.002-0.007) | 2.54E-01 | 345.61 | 9.15 | 1.06E-01 |
| Short sleep | CAD | 228 | 0.001 (-0.005-0.007) | 7.10E-01 | 251.11 | 9.6 | 1.30E-01 |
|  | MI | 226 | 0.002 (-0.004-0.008) | 5.06E-01 | 245.49 | 8.35 | 1.66E-01 |
|  | HF | 227 | 0.004 (-0.001-0.009) | 1.45E-01 | 250.05 | 9.62 | 1.30E-01 |
|  | AF | 229 | 0.002 (-0.002-0.006) | 4.04E-01 | 243.23 | 6.26 | 2.33E-01 |
|  | Stroke | 229 | 0.004 (-0.001-0.010) | 1.35E-01 | 233.52 | 2.36 | 3.87E-01 |
| Long sleep | CAD | 142 | 0.001 (-0.005-0.008) | 6.42E-01 | 174.43 | 19.17 | 2.93E-02 |
|  | MI | 140 | 0.001 (-0.005-0.007) | 6.96E-01 | 138.16 | 0 | 5.04E-01 |
|  | HF | 136 | 0.005 (-0.001-0.010) | 1.31E-01 | 163.96 | 17.66 | 4.55E-02 |
|  | AF | 143 | -0.001 (-0.005-0.004) | 7.87E-01 | 171.27 | 0.17 | 4.76E-02 |
|  | Stroke | 141 | 0.001 (-0.006-0.007) | 8.54E-01 | 154.92 | 9.63 | 1.84E-01 |
| Daytime napping | CAD | 81 | 0.009 (-0.001-0.020) | 7.90E-02 | 106.57 | 24.93 | 2.52E-02 |
|  | MI | 81 | 0.012 (0.000-0.023) | 5.80E-02 | 117.24 | **31.76** | **4.24E-03** |
|  | HF | 81 | 0.003 (-0.005-0.012) | 4.67E-01 | 101.14 | 20.9 | 5.54E-02 |
|  | AF | 79 | 0.002 (-0.007-0.010) | 6.97E-01 | 101.23 | 22.95 | 3.97E-02 |
|  | Stroke | 81 | 0.000 (-0.011-0.010) | 9.30E-01 | 107.28 | **25.43** | **2.26E-02** |
| Daytime sleepiness | CAD | 234 | 0.002 (-0.004-0.007) | 5.70E-01 | 221.23 | 0 | 7.00E-01 |
|  | MI | 234 | 0.000 (-0.007-0.006) | 9.00E-01 | 260.35 | 10.5 | 1.05E-01 |
|  | HF | 229 | 0.002 (-0.004-0.007) | 5.22E-01 | 318.92 | **28.51** | **6.52E-05** |
|  | AF | 238 | 0.001 (-0.004-0.005) | 7.00E-01 | 293.88 | 19.36 | 6.97E-03 |
|  | Stroke | 235 | 0.004 (-0.001-0.009) | 1.39E-01 | 226.43 | 0 | 6.27E-01 |
| Chronotype | CAD | 115 | -0.001 (-0.008-0.006) | 7.64E-01 | 148.69 | 23.33 | 1.61E-02 |
|  | MI | 115 | 0.001 (-0.007-0.008) | 8.45E-01 | 139.25 | 18.13 | 5.41E-02 |
|  | HF | 116 | -0.002 (-0.007-0.004) | 5.95E-01 | 146.62 | 21.57 | 2.48E-02 |
|  | AF | 116 | 0.002 (-0.003-0.008) | 4.26E-01 | 158.35 | **27.38** | **4.59E-03** |
|  | Stroke | 115 | 0.002 (-0.005-0.008) | 6.10E-01 | 127.14 | 10.33 | 1.89E-01 |
| Morning person | CAD | 93 | -0.002 (-0.009-0.006) | 6.32E-01 | 105.61 | 12.88 | 1.57E-01 |
|  | MI | 93 | -0.001 (-0.009-0.006) | 7.27E-01 | 89.13 | 0 | 5.65E-01 |
|  | HF | 94 | 0.001 (-0.005-0.008) | 6.57E-01 | 116.61 | 20.25 | 4.93E-02 |
|  | AF | 93 | 0.001 (-0.006-0.007) | 8.73E-01 | 126.58 | **27.32** | **9.81E-03** |
|  | Stroke | 93 | 0.003 (-0.005-0.010) | 5.02E-01 | 110.67 | 16.87 | 8.99E-02 |
| Snoring | CAD | 242 | 0.000 (-0.008-0.008) | 9.31E-01 | 282.97 | 14.83 | 3.28E-02 |
|  | MI | 237 | 0.003 (-0.005-0.012) | 4.41E-01 | 269.31 | 12.37 | 6.72E-02 |
|  | HF | 241 | 0.002 (-0.005-0.010) | 5.80E-01 | 287.71 | 16.58 | 1.88E-02 |
|  | AF | 235 | 0.003 (-0.003-0.009) | 3.03E-01 | 327.56 | **28.56** | **5.14E-05** |
|  | Stroke | 245 | 0.002 (-0.006-0.011) | 5.85E-01 | 259.86 | 6.1 | 2.32E-01 |
| Apnea-hypopnea index | CAD | 61 | 0.001 (-0.004-0.007) | 6.68E-01 | 73.26 | 18.1 | 1.17E-01 |
|  | MI | 60 | 0.000 (-0.006-0.006) | 9.38E-01 | 59.99 | 1.65 | 4.40E-01 |
|  | HF | 58 | 0.002 (-0.003-0.007) | 3.70E-01 | 69.9 | 18.45 | 1.17E-01 |
|  | AF | 60 | -0.001 (-0.005-0.004) | 7.23E-01 | 49.26 | 0 | 8.13E-01 |
|  | Stroke | 59 | -0.001 (-0.006-0.005) | 8.01E-01 | 73.94 | 21.56 | 7.73E-02 |

CAD, coronary artery disease; MI, myocardial infarction; HF, heart failure; AF, atrial fibrillation.

*The formula for I^2^ is (Q-DF) /Q, DF represents the degree of freedom of the Cochran’s Q statistic.

†Boldface type indicates statistically significant heterogeneity (I^2^ >25% and *P* <0.05).

## Table S6. The pleiotropy and heterogeneity for primary univariate MR analyses of sleep traits on MDD.

| Exposure | Mediator | No. SNPs | MR-Egger intercept test | | Cochran’s Q-statistic test † | | |
| --- | --- | --- | --- | --- | --- | --- | --- |
|  |  |  | Intercept (95% CI) | *P*-value | Q-statistic | I^2^, %* | *P*-value |
| Insomnia | MDD | 273 | 0.004 (0.000-0.009) | 5.10E-02 | 321.91 | 15.51 | 2.02E-02 |
| Sleep duration | MDD | 320 | -0.002 (-0.006-0.002) | 3.65E-01 | 483.49 | **34.02** | **7.22E-09** |
| Short sleep | MDD | 234 | 0.005 (0.000-0.010) | 6.00E-02 | 290.73 | 19.86 | 6.02E-03 |
| Long sleep | MDD | 147 | 0.002 (-0.004-0.008) | 5.26E-01 | 180.98 | 19.33 | 2.61E-02 |
| Daytime napping | MDD | 78 | 0.001 (-0.008-0.011) | 7.88E-01 | 123.5 | **37.65** | **6.13E-04** |
| Daytime sleepiness | MDD | 241 | 0.001 (-0.005-0.006) | 8.13E-01 | 352.97 | **32.01** | **2.74E-06** |
| Chronotype | MDD | 116 | 0.001 (-0.005-0.007) | 7.72E-01 | 150.27 | 23.47 | 1.52E-02 |
| Morning person | MDD | 94 | -0.001 (-0.008-0.005) | 7.32E-01 | 121.32 | 23.34 | 2.59E-02 |
| Snoring | MDD | 242 | 0.007 (0.000-0.014) | 6.80E-02 | 312.2 | 22.81 | 1.35E-03 |
| Apnea–hypopnea index | MDD | 61 | 0.001 (-0.004-0.006) | 7.64E-01 | 74.98 | 19.98 | 9.22E-02 |

MDD, major depressive disorder.

*The formula for I^2^ is (Q-DF) /Q, DF represents the degree of freedom of the Cochran’s Q statistic.

†Boldface type indicates statistically significant heterogeneity (I^2^ >25% and *P* <0.05).

## Table S7. The main univariate MR analyses results of the causal effects of MDD on CVDs.

| Mediator | Outcome | No. SNPs | MR-Egger intercept test | | Cochran’s Q-statistic test | | | IVW method | OR (95%CI) | *P*-value | *q*-value |
| --- | --- | --- | --- | --- | --- | --- | --- | --- | --- | --- | --- |
|  |  |  | Intercept (95% CI) | *P*-value | Q-statistic | I^2^, %* | *P*-value |  |  |  |  |
| MDD | CAD | 181 | 0.003 (-0.001-0.007) | 1.89E-01 | 214.33 | 16.02 | 4.09E-02 | fixed-effect IVW | 1.06 (1.02-1.10) | 1.69E-03 | 3.65E-03** |
| MDD | MI | 178 | 0.003 (-0.002-0.008) | 2.00E-01 | 201.86 | 12.32 | 9.69E-02 | fixed-effect IVW | 1.06 (1.02-1.11) | 2.19E-03 | 3.65E-03** |
| MDD | HF | 176 | 0.001 (-0.002-0.005) | 4.79E-01 | 203.46 | 6.93 | 6.93E-02 | fixed-effect IVW | 1.07 (1.03-1.10) | 3.22E-05 | 1.61E-04** |
| MDD | AF | 182 | 0.000 (-0.003-0.003) | 9.01E-01 | 194.85 | 7.11 | 2.28E-01 | fixed-effect IVW | 1.01 (0.98-1.04) | 4.58E-01 | 4.58E-01 |
| MDD | Stroke | 184 | 0.000 (-0.004-0.004) | 8.82E-01 | 187.92 | 2.62 | 3.86E-01 | fixed-effect IVW | 1.05 (1.01-1.08) | 1.10E-02 | 1.38E-02** |

MDD, major depressive disorder; CAD, coronary artery disease; MI, myocardial infarction; HF, heart failure; AF, atrial fibrillation.

*The formula for I^2^ is (Q-DF) /Q, DF represents the degree of freedom of the Cochran’s Q statistic.

**Indicates significant evidence (*q*-value <0.05) after correction for multiple testing estimated by the FDR method.

## Table S8. The pleiotropy and heterogeneity test results of effects of MDD on each CVDs after adjusting for each sleep trait.

| Mediator | Adjustment factors | Outcome | No. SNPs | Conditional F-statistics‡ | | Multivariable MR-Egger intercept test § | | Multivariable heterogeneity test | | |
| --- | --- | --- | --- | --- | --- | --- | --- | --- | --- | --- |
|  |  |  |  | F_1_ | F_2_ | Intercept (95% CI) | *P*-value | Q-statistic | I^2^, %* | *P*-value |
| MDD | Insomnia | CAD | 451 | 13.79 | 8.06 | 0.000 (-0.002-0.002) | 7.63E-01 | 510.56 | 12.25 | 2.16E-02 |
|  |  | MI | 446 | 13.78 | 7.98 | 0.000 (-0.002-0.002) | 7.41E-01 | 485.09 | 8.68 | 8.17E-02 |
|  |  | HF | 443 | 13.83 | 8.07 | -0.001 (-0.003-0.001) | 4.81E-01 | 530.19 | 17.01 | 2.01E-03 |
|  |  | AF | 459 | 13.82 | 7.96 | 0.000 (-0.002-0.002) | 6.18E-01 | 576.16 | 20.86 | 1.09E-04 |
|  |  | Stroke | 455 | 13.64 | 7.99 | 0.000 (-0.002-0.002) | 8.01E-01 | 563.04 | 19.72 | 2.81E-04 |
| MDD | Sleep duration | CAD | 498 | 15.73 | 8.18 | 0.001 (-0.001-0.003) | 3.73E-01 | 558.03 | 11.29 | 2.59E-02 |
|  |  | MI | 496 | 15.83 | 8.11 | 0.000 (-0.002-0.002) | 7.22E-01 | 527.79 | 6.59 | 1.35E-01 |
|  |  | HF | 490 | 15.79 | 8.12 | 0.000 (-0.002-0.002) | 7.03E-01 | 527.74 | 7.72 | 9.82E-02 |
|  |  | AF | 497 | 15.55 | 8.2 | 0.000 (-0.002-0.002) | 7.87E-01 | 575.20 | 14.12 | 6.66E-03 |
|  |  | Stroke | 499 | 15.53 | 8.26 | 0.001 (-0.001-0.003) | 2.11E-01 | 528.70 | 6.19 | 1.50E-01 |
| MDD | Short sleep | CAD | 409 | 12.44 | 9.4 | 0.001 (-0.001-0.003) | 3.09E-01 | 457.05 | 11.17 | 4.05E-02 |
|  |  | MI | 404 | 12.46 | 9.36 | 0.001 (-0.001-0.003) | 4.98E-01 | 438.47 | 8.55 | 9.55E-02 |
|  |  | HF | 403 | 12.36 | 9.05 | 0.000 (-0.002-0.002) | 9.85E-01 | 447.44 | 10.6 | 5.07E-02 |
|  |  | AF | 411 | 12.45 | 9.5 | 0.001 (-0.001-0.003) | 2.52E-01 | 437.48 | 6.74 | 1.51E-01 |
|  |  | Stroke | 413 | 12.29 | 9.29 | 0.000 (-0.002-0.002) | 9.27E-01 | 418.51 | 2.03 | 3.75E-01 |
| MDD | Long sleep | CAD | 323 | 9.75 | 11.32 | 0.001 (-0.001-0.003) | 3.24E-01 | 384.90 | 16.86 | 7.43E-03 |
|  |  | MI | 318 | 9.76 | 11.29 | 0.001 (-0.001-0.003) | 4.52E-01 | 338.65 | 6.98 | 1.72E-01 |
|  |  | HF | 312 | 9.54 | 11.42 | 0.001 (-0.001-0.003) | 4.58E-01 | 368.47 | 16.14 | 1.13E-02 |
|  |  | AF | 325 | 10.42 | 12.1 | 0.000 (-0.002-0.002) | 8.54E-01 | 365.64 | 11.94 | 4.71E-02 |
|  |  | Stroke | 325 | 9.71 | 11.56 | 0.001 (-0.001-0.003) | 3.21E-01 | 341.63 | 5.75 | 2.16E-01 |
| MDD | Daytime napping | CAD | 261 | 15.5 | 13.97 | 0.001 (-0.001-0.003) | 6.59E-01 | 321.76 | 19.82 | 4.23E-03 |
|  |  | MI | 258 | 15.58 | 13.82 | 0.001 (-0.003-0.005) | 3.41E-01 | 319.17 | 20.1 | 3.89E-03 |
|  |  | HF | 256 | 15.67 | 13.76 | 0.000 (-0.002-0.002) | 8.31E-01 | 300.86 | 15.91 | 2.09E-02 |
|  |  | AF | 260 | 14.63 | 14.32 | 0.000 (-0.002-0.002) | 7.61E-01 | 296.76 | 13.4 | 4.46E-02 |
|  |  | Stroke | 264 | 15.34 | 14.23 | -0.001 (-0.003-0.001) | 4.91E-01 | 290.77 | 10.24 | 9.93E-02 |
| MDD | Daytime sleepiness | CAD | 415 | 12.95 | 9.3 | -0.001 (-0.003-0.001) | 4.21E-01 | 436.47 | 5.61 | 1.95E-01 |
|  |  | MI | 412 | 13.05 | 9.23 | -0.001 (-0.003-0.001) | 4.26E-01 | 462.87 | 11.64 | 3.36E-02 |
|  |  | HF | 405 | 13.01 | 9.26 | 0.000 (-0.002-0.002) | 9.47E-01 | 516.77 | 22.21 | 9.23E-05 |
|  |  | AF | 420 | 12.94 | 9.28 | -0.002 (-0.004-0.000) | 9.60E-02 | 486.90 | 14.36 | 1.02E-02 |
|  |  | Stroke | 419 | 12.99 | 9.36 | 0.000 (-0.002-0.002) | 7.42E-01 | 412.88 | 0 | 5.34E-01 |
| MDD | Chronotype | CAD | 296 | 19.81 | 12.59 | 0.000 (-0.002-0.002) | 7.86E-01 | 362.10 | 19.08 | 3.64E-03 |
|  |  | MI | 293 | 20.01 | 12.52 | 0.002 (0.000-0.004) | 1.31E-01 | 340.36 | 14.8 | 2.23E-02 |
|  |  | HF | 292 | 20.43 | 12.39 | 0.001 (-0.001-0.003) | 6.05E-01 | 347.44 | 16.82 | 1.04E-02 |
|  |  | AF | 298 | 20.07 | 12.64 | 0.000 (-0.002-0.002) | 9.19E-01 | 353.17 | 16.47 | 1.13E-02 |
|  |  | Stroke | 299 | 19.85 | 12.67 | -0.001 (-0.003-0.001) | 3.07E-01 | 315.46 | 6.17 | 2.09E-01 |
| MDD | Morning person | CAD | 274 | 16.88 | 13.32 | 0.000 (-0.002-0.002) | 9.84E-01 | 319.82 | 15.26 | 2.22E-02 |
|  |  | MI | 271 | 17.06 | 13.26 | 0.001 (-0.003-0.005) | 6.50E-01 | 289.75 | 7.51 | 1.73E-01 |
|  |  | HF | 270 | 17.29 | 12.8 | 0.002 (0.000-0.004) | 1.00E-01 | 318.15 | 16.08 | 1.72E-02 |
|  |  | AF | 275 | 17.02 | 12.89 | 0.000 (-0.002-0.002) | 8.95E-01 | 320.91 | 15.24 | 2.21E-02 |
|  |  | Stroke | 277 | 16.89 | 13.32 | 0.001 (-0.001-0.003) | 6.27E-01 | 295.57 | 7.3 | 1.77E-01 |
| 0MDD | Snoring | CAD | 423 | 13.21 | 9.19 | 0.002 (0.000-0.004) | 1.12E-01 | 496.86 | 15.47 | 5.73E-03 |
|  |  | MI | 415 | 13.22 | 9.21 | 0.002 (0.000-0.004) | 8.80E-02 | 471.74 | 12.66 | 2.22E-02 |
|  |  | HF | 417 | 13.32 | 9.09 | 0.001 (-0.001-0.003) | 3.91E-01 | 489.96 | 15.5 | 5.93E-03 |
|  |  | AF | 417 | 13 | 9.37 | -0.001 (-0.003-0.001) | 4.99E-01 | 522.25 | 20.73 | 2.33E-04 |
|  |  | Stroke | 429 | 13.22 | 9.22 | -0.001 (-0.003-0.001) | 4.84E-01 | 447.99 | 4.91 | 2.23E-01 |
| MDD | Apnea–hypopnea index | CAD | 242 | 5.37 | 14.96 | 0.000 (-0.002-0.002) | 9.92E-01 | 285.77 | 16.37 | 2.05E-02 |
|  |  | MI | 238 | 5.36 | 14.92 | 0.001 (-0.001-0.003) | 3.27E-01 | 260.70 | 9.86 | 1.20E-01 |
|  |  | HF | 234 | 5.32 | 15.03 | -0.001 (-0.003-0.001) | 5.02E-01 | 272.40 | 15.2 | 3.20E-02 |
|  |  | AF | 242 | 5.29 | 15.11 | **0.003 (0.001-0.005)** | **3.00E-03** | 243.59 | 1.88 | 4.06E-01 |
|  |  | Stroke | 243 | 5.21 | 15.13 | 0.001 (-0.001-0.003) | 6.65E-01 | 262.73 | 8.65 | 1.50E-01 |

MDD, major depressive disorder; CAD, coronary artery disease; MI, myocardial infarction; HF, heart failure; AF, atrial fibrillation.

*DF represents the degree of freedom of the Cochran’s Q statistic.

†The formula for I^2^ is (Q-DF) /Q.

‡F_1_ for adjustment factors (exposure), F_2_ for MDD

§Boldface type indicates statistically significant pleiotropy (*P* <0.05).

## Table S9. The complementary MR analysis results of the causal effects of sleep traits on CVDs.

| Exposure | Outcome | No. SNPs | Maximum likelihood | | MR Egger | | IVW-SIMEXS (weighted) | | Weighted median | | Penalised weighted median | |
| --- | --- | --- | --- | --- | --- | --- | --- | --- | --- | --- | --- | --- |
|  |  |  | OR (95% CI) | *P*-value | OR (95% CI) | *P*-value | OR (95% CI) | *P*-value | OR (95% CI) | *P*-value | OR (95% CI) | *P*-value |
| Insomnia | CAD | 270 | 1.49 (1.24-1.78) | 1.59E-05 | 1.2 (0.63-2.25) | 5.81E-01 | 1.49 (1.23-1.81) | 5.87E-05 | 1.51 (1.16-1.97) | 2.39E-03 | 1.51 (1.16-1.98) | 2.32E-03 |
|  | MI | 268 | 1.58 (1.29-1.92) | 8.89E-06 | 1.53 (0.76-3.05) | 2.32E-01 | 1.58 (1.28-1.95) | 2.84E-05 | 1.58 (1.18-2.1) | 2.05E-03 | 1.58 (1.16-2.14) | 3.36E-03 |
|  | HF | 267 | 1.32 (1.14-1.53) | 2.49E-04 | 2.16 (1.27-3.67) | 4.60E-03 | 1.33 (1.12-1.57) | 1.01E-03 | 1.36 (1.08-1.71) | 9.41E-03 | 1.36 (1.08-1.71) | 7.92E-03 |
|  | AF | 277 | 1.25 (1.09-1.43) | 1.70E-03 | 0.84 (0.5-1.41) | 5.10E-01 | 1.25 (1.06-1.47) | 7.77E-03 | 1.05 (0.86-1.29) | 6.19E-01 | 1.05 (0.86-1.29) | 6.20E-01 |
|  | Stroke | 271 | 1.27 (1.06-1.51) | 8.81E-03 | 1.12 (0.56-2.21) | 7.52E-01 | 1.27 (1.02-1.57) | 3.13E-02 | 1.39 (1.04-1.84) | 2.48E-02 | 1.42 (1.08-1.86) | 1.24E-02 |
| Sleep duration | CAD | 317 | 0.88 (0.8-0.97) | 1.10E-02 | 0.79 (0.57-1.08) | 1.38E-01 | 0.88 (0.79-0.98) | 1.75E-02 | 0.86 (0.73-1) | 5.21E-02 | 0.86 (0.73-1) | 5.22E-02 |
|  | MI | 318 | 0.89 (0.8-0.99) | 3.45E-02 | 0.99 (0.71-1.39) | 9.63E-01 | 0.89 (0.79-0.99) | 3.70E-02 | 0.94 (0.81-1.11) | 4.75E-01 | 0.94 (0.8-1.12) | 5.02E-01 |
|  | HF | 314 | 0.9 (0.83-0.98) | 1.23E-02 | 0.99 (0.76-1.29) | 9.35E-01 | 0.9 (0.82-0.98) | 1.44E-02 | 0.89 (0.79-1) | 4.98E-02 | 0.89 (0.78-1.02) | 8.51E-02 |
|  | AF | 315 | 0.94 (0.87-1.01) | 1.09E-01 | 1.09 (0.84-1.41) | 5.31E-01 | 0.94 (0.86-1.02) | 1.00E-01 | 0.99 (0.88-1.12) | 8.80E-01 | 0.99 (0.88-1.12) | 9.13E-01 |
|  | Stroke | 315 | 0.99 (0.9-1.09) | 8.83E-01 | 0.83 (0.59-1.15) | 2.59E-01 | 0.99 (0.9-1.1) | 8.93E-01 | 1 (0.87-1.16) | 9.96E-01 | 1.01 (0.87-1.17) | 9.44E-01 |
| Short sleep | CAD | 228 | 1.58 (1.18-2.11) | 2.21E-03 | 1.29 (0.47-3.55) | 6.27E-01 | 1.58 (1.16-2.16) | 4.41E-03 | 1.73 (1.12-2.69) | 1.44E-02 | 1.73 (1.13-2.66) | 1.25E-02 |
|  | MI | 226 | 1.51 (1.1-2.09) | 1.21E-02 | 1.04 (0.34-3.19) | 9.47E-01 | 1.53 (1.09-2.17) | 1.56E-02 | 1.5 (0.92-2.43) | 1.04E-01 | 1.5 (0.95-2.37) | 8.38E-02 |
|  | HF | 227 | 1.4 (1.1-1.78) | 6.43E-03 | 0.74 (0.3-1.78) | 4.96E-01 | 1.4 (1.08-1.81) | 1.10E-02 | 1.33 (0.94-1.89) | 1.06E-01 | 1.33 (0.95-1.87) | 1.01E-01 |
|  | AF | 229 | 1.1 (0.88-1.38) | 3.94E-01 | 0.8 (0.38-1.72) | 5.77E-01 | 1.11 (0.87-1.4) | 4.04E-01 | 1.15 (0.83-1.6) | 3.87E-01 | 1.15 (0.84-1.59) | 3.79E-01 |
|  | Stroke | 229 | 1.18 (0.88-1.57) | 2.64E-01 | 0.56 (0.2-1.53) | 2.58E-01 | 1.18 (0.87-1.59) | 2.89E-01 | 1.3 (0.87-1.96) | 2.05E-01 | 1.31 (0.89-1.94) | 1.77E-01 |
| Long sleep | CAD | 142 | 1.58 (0.96-2.59) | 7.05E-02 | 1.16 (0.31-4.35) | 8.25E-01 | 1.59 (0.91-2.79) | 1.05E-01 | 1.96 (0.92-4.2) | 8.30E-02 | 2.1 (0.95-4.63) | 6.76E-02 |
|  | MI | 140 | 1.33 (0.77-2.3) | 3.10E-01 | 1.04 (0.27-3.97) | 9.59E-01 | 1.34 (0.77-2.35) | 3.02E-01 | 1.91 (0.82-4.46) | 1.33E-01 | 1.95 (0.83-4.55) | 1.24E-01 |
|  | HF | 136 | 1.36 (0.89-2.09) | 1.58E-01 | 0.52 (0.14-1.92) | 3.27E-01 | 1.36 (0.84-2.19) | 2.12E-01 | 1.12 (0.59-2.1) | 7.31E-01 | 1.12 (0.59-2.1) | 7.32E-01 |
|  | AF | 144 | 1.33 (0.91-1.95) | 1.43E-01 | 1.5 (0.52-4.3) | 4.51E-01 | 1.34 (0.87-2.05) | 1.88E-01 | 1.28 (0.73-2.25) | 3.86E-01 | 1.15 (0.67-1.98) | 6.14E-01 |
|  | Stroke | 141 | 1.13 (0.68-1.87) | 6.48E-01 | 0.98 (0.21-4.52) | 9.77E-01 | 1.13 (0.65-1.94) | 6.68E-01 | 1.39 (0.67-2.89) | 3.72E-01 | 1.43 (0.7-2.96) | 3.28E-01 |
| Daytime napping | CAD | 81 | 0.91 (0.7-1.19) | 5.03E-01 | 0.34 (0.11-1.05) | 6.55E-02 | 0.91 (0.67-1.24) | 5.60E-01 | 0.84 (0.56-1.25) | 3.87E-01 | 0.84 (0.57-1.24) | 3.73E-01 |
|  | MI | 81 | 0.81 (0.6-1.09) | 1.58E-01 | 0.24 (0.06-0.87) | 3.31E-02 | 0.81 (0.57-1.16) | 2.52E-01 | 0.79 (0.5-1.24) | 2.98E-01 | 0.79 (0.51-1.22) | 2.84E-01 |
|  | HF | 81 | 1.4 (1.12-1.76) | 3.13E-03 | 0.99 (0.38-2.56) | 9.80E-01 | 1.4 (1.09-1.81) | 1.15E-02 | 1.11 (0.79-1.56) | 5.44E-01 | 1.07 (0.75-1.53) | 6.94E-01 |
|  | AF | 79 | 1.28 (1.04-1.57) | 1.93E-02 | 1.06 (0.41-2.73) | 9.07E-01 | 1.27 (1-1.61) | 4.98E-02 | 1.43 (1.06-1.94) | 1.93E-02 | 1.45 (1.06-1.99) | 2.01E-02 |
|  | Stroke | 81 | 1.05 (0.81-1.37) | 7.03E-01 | 1.11 (0.35-3.49) | 8.65E-01 | 1.05 (0.77-1.43) | 7.51E-01 | 1 (0.66-1.52) | 1.00E+00 | 1 (0.66-1.51) | 1.00E+00 |
| Daytime sleepiness | CAD | 234 | 1.41 (1.09-1.83) | 9.91E-03 | 1.11 (0.48-2.56) | 8.14E-01 | 1.41 (1.09-1.84) | 9.84E-03 | 1.45 (0.99-2.12) | 5.59E-02 | 1.46 (1-2.13) | 5.19E-02 |
|  | MI | 234 | 1.19 (0.89-1.59) | 2.31E-01 | 1.26 (0.47-3.38) | 6.47E-01 | 1.19 (0.87-1.63) | 2.73E-01 | 1.1 (0.71-1.69) | 6.79E-01 | 1.06 (0.7-1.62) | 7.71E-01 |
|  | HF | 229 | 1.14 (0.91-1.42) | 2.58E-01 | 0.86 (0.35-2.08) | 7.33E-01 | 1.14 (0.88-1.49) | 3.22E-01 | 1.2 (0.85-1.7) | 3.10E-01 | 1.17 (0.83-1.66) | 3.73E-01 |
|  | AF | 238 | 1.1 (0.9-1.35) | 3.50E-01 | 0.96 (0.46-1.98) | 9.07E-01 | 1.1 (0.88-1.38) | 4.02E-01 | 1.33 (0.99-1.78) | 5.59E-02 | 1.39 (1.03-1.88) | 3.26E-02 |
|  | Stroke | 235 | 1 (0.78-1.3) | 9.79E-01 | 0.54 (0.22-1.27) | 1.59E-01 | 1 (0.77-1.29) | 9.98E-01 | 0.97 (0.67-1.41) | 8.92E-01 | 0.97 (0.67-1.41) | 8.90E-01 |
| Chronotype | CAD | 115 | 0.99 (0.9-1.09) | 8.47E-01 | 1.04 (0.75-1.45) | 8.19E-01 | 0.99 (0.89-1.11) | 8.87E-01 | 1.03 (0.89-1.19) | 6.92E-01 | 1.06 (0.92-1.22) | 4.39E-01 |
|  | MI | 115 | 1 (0.9-1.11) | 9.58E-01 | 0.96 (0.68-1.37) | 8.41E-01 | 1 (0.89-1.12) | 9.56E-01 | 0.97 (0.83-1.13) | 6.57E-01 | 0.96 (0.82-1.12) | 6.09E-01 |
|  | HF | 116 | 1.05 (0.97-1.14) | 2.15E-01 | 1.12 (0.87-1.45) | 3.84E-01 | 1.05 (0.96-1.15) | 2.78E-01 | 1.14 (1.01-1.29) | 3.30E-02 | 1.14 (1.01-1.29) | 2.98E-02 |
|  | AF | 116 | 1.06 (0.98-1.14) | 1.44E-01 | 0.96 (0.75-1.23) | 7.39E-01 | 1.06 (0.97-1.15) | 2.17E-01 | 1.01 (0.91-1.13) | 8.47E-01 | 0.98 (0.87-1.1) | 7.35E-01 |
|  | Stroke | 115 | 1.04 (0.94-1.14) | 4.48E-01 | 0.96 (0.72-1.29) | 8.09E-01 | 1.04 (0.94-1.14) | 4.79E-01 | 1.05 (0.91-1.21) | 4.98E-01 | 1.06 (0.93-1.2) | 4.14E-01 |
| Morning person | CAD | 93 | 1.09 (0.83-1.43) | 5.51E-01 | 1.33 (0.55-3.22) | 5.28E-01 | 1.08 (0.8-1.46) | 5.96E-01 | 1.25 (0.84-1.88) | 2.71E-01 | 1.28 (0.87-1.88) | 2.06E-01 |
|  | MI | 93 | 1.04 (0.77-1.41) | 8.06E-01 | 1.21 (0.49-3.01) | 6.82E-01 | 1.04 (0.77-1.41) | 8.05E-01 | 0.97 (0.62-1.5) | 8.81E-01 | 0.97 (0.61-1.53) | 8.85E-01 |
|  | HF | 94 | 1.13 (0.9-1.41) | 3.06E-01 | 0.96 (0.45-2.02) | 9.06E-01 | 1.12 (0.87-1.45) | 3.76E-01 | 1.16 (0.82-1.64) | 3.97E-01 | 1.06 (0.75-1.49) | 7.32E-01 |
|  | AF | 93 | 1.05 (0.85-1.3) | 6.18E-01 | 0.99 (0.48-2.07) | 9.89E-01 | 1.05 (0.82-1.35) | 6.88E-01 | 0.93 (0.67-1.3) | 6.89E-01 | 0.9 (0.65-1.24) | 5.25E-01 |
|  | Stroke | 93 | 0.94 (0.71-1.23) | 6.33E-01 | 0.7 (0.29-1.71) | 4.35E-01 | 0.94 (0.69-1.26) | 6.68E-01 | 1.2 (0.8-1.79) | 3.75E-01 | 1.22 (0.81-1.84) | 3.31E-01 |
| Snoring | CAD | 242 | 1.04 (0.99-1.1) | 1.20E-01 | 1 (0.83-1.21) | 9.86E-01 | 1.04 (0.98-1.11) | 1.59E-01 | 1.01 (0.93-1.09) | 8.03E-01 | 0.99 (0.92-1.07) | 8.79E-01 |
|  | MI | 237 | 1 (0.95-1.06) | 9.24E-01 | 1.01 (0.82-1.24) | 9.20E-01 | 1 (0.94-1.07) | 9.47E-01 | 0.97 (0.89-1.06) | 4.68E-01 | 0.97 (0.89-1.06) | 4.68E-01 |
|  | HF | 241 | 1.03 (0.98-1.07) | 2.61E-01 | 0.96 (0.82-1.12) | 5.81E-01 | 1.03 (0.98-1.08) | 3.18E-01 | 1.03 (0.97-1.1) | 3.37E-01 | 1.03 (0.96-1.1) | 3.94E-01 |
|  | AF | 235 | 1.01 (0.97-1.05) | 7.03E-01 | 1.04 (0.89-1.21) | 6.63E-01 | 1.01 (0.96-1.06) | 7.72E-01 | 1.01 (0.95-1.07) | 8.60E-01 | 1.01 (0.95-1.07) | 8.35E-01 |
|  | Stroke | 245 | 1.01 (0.96-1.06) | 8.11E-01 | 1.03 (0.86-1.23) | 7.58E-01 | 1.01 (0.95-1.06) | 8.24E-01 | 0.98 (0.91-1.06) | 6.98E-01 | 0.98 (0.91-1.06) | 6.85E-01 |
| Apnea–hypopnea index | CAD | 61 | 1.02 (0.99-1.05) | 1.88E-01 | 1.02 (0.96-1.09) | 5.17E-01 | 1.02 (0.99-1.05) | 2.45E-01 | 1.02 (0.98-1.06) | 4.09E-01 | 1.02 (0.97-1.06) | 4.33E-01 |
|  | MI | 60 | 1.01 (0.98-1.04) | 5.57E-01 | 0.99 (0.92-1.05) | 6.85E-01 | 1.01 (0.98-1.04) | 5.72E-01 | 1.02 (0.97-1.07) | 4.64E-01 | 1.02 (0.97-1.07) | 4.40E-01 |
|  | HF | 58 | 1.01 (0.99-1.04) | 2.84E-01 | 1 (0.94-1.06) | 9.53E-01 | 1.01 (0.99-1.04) | 3.26E-01 | 1.01 (0.97-1.05) | 5.61E-01 | 1.01 (0.97-1.05) | 5.58E-01 |
|  | AF | 60 | 0.99 (0.97-1.01) | 4.38E-01 | 0.97 (0.93-1.02) | 2.01E-01 | 0.99 (0.97-1.01) | 3.80E-01 | 1 (0.97-1.03) | 9.59E-01 | 1 (0.97-1.03) | 9.05E-01 |
|  | Stroke | 59 | 1.03 (1-1.06) | 7.34E-02 | 1.01 (0.94-1.08) | 7.98E-01 | 1.03 (0.99-1.06) | 1.04E-01 | 1.03 (0.99-1.08) | 1.82E-01 | 1.03 (0.99-1.07) | 1.90E-01 |

CAD, coronary artery disease; MI, myocardial infarction; HF, heart failure; AF, atrial fibrillation. The OR represented the effect of genetically predicted per unit increase in each sleep trait.

## Table S10. The complementary MR analysis results of the causal effects of sleep traits on MDD.

| Exposure | Mediator | No. SNPs | Maximum likelihood | | MR Egger | | IVW-SIMEXS (weighted) | | Weighted median | | Penalised weidhted median | |
| --- | --- | --- | --- | --- | --- | --- | --- | --- | --- | --- | --- | --- |
|  |  |  | OR (95% CI) | *P*-value | OR (95% CI) | *P*-value | OR (95% CI) | *P*-value | OR (95% CI) | *P*-value | OR (95% CI) | *P*-value |
| Insomnia | MDD | 273 | 2.12 (1.82-2.47) | 7.74E-22 | 1.26 (0.74-2.12) | 3.97E-01 | 2.14 (1.81-2.54) | <2E-16 | 1.78 (1.42-2.25) | 9.29E-07 | 1.79 (1.42-2.26) | 8.57E-07 |
| Sleep duration | MDD | 320 | 0.84 (0.77-0.91) | 3.42E-05 | 0.97 (0.7-1.35) | 8.76E-01 | 0.84 (0.76-0.93) | 1.12E-03 | 0.87 (0.76-1) | 4.63E-02 | 0.87 (0.76-0.99) | 3.69E-02 |
| Short sleep | MDD | 234 | 1.88 (1.47-2.41) | 5.91E-07 | 0.79 (0.32-1.96) | 6.09E-01 | 1.88 (1.42-2.48) | 1.82E-05 | 1.79 (1.25-2.56) | 1.42E-03 | 1.84 (1.29-2.62) | 7.65E-04 |
| Long sleep | MDD | 147 | 1.78 (1.16-2.74) | 8.02E-03 | 1.18 (0.33-4.27) | 7.99E-01 | 1.8 (1.11-2.93) | 1.81E-02 | 1.59 (0.86-2.93) | 1.39E-01 | 1.59 (0.85-2.96) | 1.46E-01 |
| Daytime napping | MDD | 78 | 1.43 (1.14-1.81) | 2.23E-03 | 1.24 (0.44-3.5) | 6.90E-01 | 1.43 (1.06-1.91) | 2.07E-02 | 1.47 (1.03-2.09) | 3.32E-02 | 1.58 (1.1-2.27) | 1.32E-02 |
| Daytime sleepiness | MDD | 241 | 1.7 (1.36-2.13) | 3.00E-06 | 1.51 (0.64-3.55) | 3.45E-01 | 1.7 (1.29-2.24) | 1.76E-04 | 1.68 (1.19-2.36) | 2.93E-03 | 1.69 (1.21-2.36) | 2.13E-03 |
| Chronotype | MDD | 116 | 0.91 (0.84-0.98) | 1.67E-02 | 0.87 (0.67-1.14) | 3.19E-01 | 0.91 (0.83-0.99) | 3.63E-02 | 0.89 (0.79-1.01) | 7.06E-02 | 0.89 (0.79-1.01) | 6.82E-02 |
| Morning person | MDD | 94 | 0.75 (0.59-0.94) | 1.29E-02 | 0.84 (0.39-1.81) | 6.58E-01 | 0.73 (0.56-0.96) | 2.60E-02 | 0.74 (0.52-1.06) | 9.75E-02 | 0.74 (0.52-1.05) | 9.10E-02 |
| Snoring | MDD | 242 | 0.97 (0.93-1.02) | 2.11E-01 | 0.95 (0.8-1.12) | 5.29E-01 | 0.97 (0.92-1.02) | 2.52E-01 | 0.9 (0.84-0.96) | 1.46E-03 | 0.89 (0.83-0.95) | 2.99E-04 |
| Apnea–hypopnea index | MDD | 61 | 1.01 (0.99-1.04) | 3.69E-01 | 0.97 (0.91-1.02) | 2.24E-01 | 1.01 (0.98-1.04) | 4.22E-01 | 1 (0.96-1.03) | 7.95E-01 | 0.99 (0.96-1.03) | 7.67E-01 |

MDD, major depressive disorder. The OR represented the effect of genetically predicted per unit increase in each sleep trait.

## Table S11. The complementary MR analysis results of the causal effects of MDD on CVDs.

| Mediator | Outcome | No. SNPs | Maximum likelihood | | MR Egger | | IVW-SIMEXS (weighted) | | Weighted median | | Penalised weidhted median | |
| --- | --- | --- | --- | --- | --- | --- | --- | --- | --- | --- | --- | --- |
|  |  |  | OR (95% CI) | *P*-value | OR (95% CI) | *P*-value | OR (95% CI) | *P*-value | OR (95% CI) | *P*-value | OR (95% CI) | *P*-value |
| MDD | CAD | 181 | 1.06 (1.02-1.1) | 2.09E-03 | 1.01 (0.94-1.09) | 7.71E-01 | 1.06 (1.02-1.11) | 5.53E-03 | 1.05 (0.99-1.11) | 1.21E-01 | 1.05 (0.99-1.11) | 1.17E-01 |
| MDD | MI | 178 | 1.07 (1.02-1.11) | 2.62E-03 | 1.01 (0.93-1.1) | 7.60E-01 | 1.07 (1.02-1.12) | 5.27E-03 | 1.03 (0.97-1.1) | 3.54E-01 | 1.03 (0.97-1.1) | 3.51E-01 |
| MDD | HF | 176 | 1.07 (1.04-1.1) | 4.30E-05 | 1.04 (0.98-1.12) | 2.03E-01 | 1.07 (1.03-1.11) | 1.66E-04 | 1.05 (0.99-1.1) | 1.14E-01 | 1.04 (0.99-1.11) | 1.36E-01 |
| MDD | AF | 182 | 1.01 (0.98-1.04) | 4.60E-01 | 1.01 (0.95-1.06) | 7.98E-01 | 1.01 (0.98-1.04) | 4.73E-01 | 1.02 (0.97-1.07) | 4.36E-01 | 1.02 (0.97-1.07) | 4.12E-01 |
| MDD | Stroke | 184 | 1.05 (1.01-1.09) | 1.15E-02 | 1.05 (0.98-1.13) | 1.77E-01 | 1.05 (1.01-1.09) | 1.25E-02 | 1.04 (0.99-1.1) | 1.43E-01 | 1.04 (0.98-1.1) | 1.64E-01 |

MDD, major depressive disorder; CAD, coronary artery disease; MI, myocardial infarction; HF, heart failure; AF, atrial fibrillation. The OR represented the effect of genetically predicted per log odds increase in MDD.

## Table S12. Verification results of potentially weak instrumental bias in causal effects of sleep traits on CVDs.

| Exposure | Outcome | Number of SNPs | Radial IVW | | |
| --- | --- | --- | --- | --- | --- |
|  |  |  | OR (95% CI) | *P*-value | *q*-value |
| Insomnia | CAD | 270 | 1.47 (1.22-1.77) | 4.59E-05 | 1.15E-03 |
|  | MI | 268 | 1.55 (1.27-1.9) | 1.91E-05 | 9.55E-04 |
|  | HF | 267 | 1.31 (1.12-1.54) | 9.65E-04 | 1.61E-02 |
|  | AF | 277 | 1.24 (1.06-1.45) | 7.96E-03 | 6.25E-02 |
|  | Stroke | 271 | 1.26 (1.03-1.54) | 2.68E-02 | 1.12E-01 |
| Sleep duration | CAD | 317 | 0.88 (0.8-0.98) | 1.56E-02 | 7.50E-02 |
|  | MI | 318 | 0.89 (0.8-0.99) | 3.90E-02 | 1.49E-01 |
|  | HF | 314 | 0.9 (0.83-0.98) | 1.46E-02 | 7.50E-02 |
|  | AF | 315 | 0.94 (0.87-1.02) | 1.48E-01 | 4.35E-01 |
|  | Stroke | 315 | 0.99 (0.9-1.1) | 8.88E-01 | 9.45E-01 |
| Short sleep | CAD | 228 | 1.55 (1.15-2.09) | 4.11E-03 | 5.14E-02 |
|  | MI | 226 | 1.5 (1.08-2.08) | 1.65E-02 | 7.50E-02 |
|  | HF | 227 | 1.38 (1.08-1.77) | 1.00E-02 | 6.25E-02 |
|  | AF | 229 | 1.1 (0.88-1.37) | 4.13E-01 | 6.07E-01 |
|  | Stroke | 229 | 1.17 (0.88-1.55) | 2.76E-01 | 5.31E-01 |
| Long sleep | CAD | 142 | 1.55 (0.91-2.64) | 1.10E-01 | 3.44E-01 |
|  | MI | 140 | 1.32 (0.78-2.26) | 3.01E-01 | 5.46E-01 |
|  | HF | 136 | 1.35 (0.85-2.12) | 2.03E-01 | 5.00E-01 |
|  | AF | 143 | 1.31 (0.87-1.97) | 1.93E-01 | 5.00E-01 |
|  | Stroke | 141 | 1.12 (0.67-1.88) | 6.69E-01 | 8.29E-01 |
| Daytime napping | CAD | 81 | 0.92 (0.68-1.24) | 5.66E-01 | 7.68E-01 |
|  | MI | 81 | 0.81 (0.57-1.15) | 2.47E-01 | 5.31E-01 |
|  | HF | 81 | 1.39 (1.08-1.79) | 9.40E-03 | 6.25E-02 |
|  | AF | 79 | 1.27 (1.01-1.6) | 4.16E-02 | 1.49E-01 |
|  | Stroke | 81 | 1.05 (0.78-1.42) | 7.41E-01 | 8.69E-01 |
| Daytime sleepiness | CAD | 234 | 1.39 (1.09-1.79) | 8.79E-03 | 6.25E-02 |
|  | MI | 234 | 1.19 (0.88-1.6) | 2.63E-01 | 5.31E-01 |
|  | HF | 229 | 1.13 (0.88-1.46) | 3.39E-01 | 5.65E-01 |
|  | AF | 238 | 1.1 (0.88-1.36) | 4.02E-01 | 6.07E-01 |
|  | Stroke | 235 | 1 (0.78-1.28) | 9.79E-01 | 9.79E-01 |
| Chronotype | CAD | 115 | 0.99 (0.89-1.1) | 8.66E-01 | 9.41E-01 |
|  | MI | 115 | 1 (0.89-1.12) | 9.61E-01 | 9.79E-01 |
|  | HF | 116 | 1.05 (0.96-1.15) | 2.76E-01 | 5.31E-01 |
|  | AF | 116 | 1.06 (0.97-1.15) | 2.10E-01 | 5.00E-01 |
|  | Stroke | 115 | 1.04 (0.94-1.14) | 4.76E-01 | 6.80E-01 |
| Morning person | CAD | 93 | 1.08 (0.81-1.45) | 5.88E-01 | 7.74E-01 |
|  | MI | 93 | 1.04 (0.77-1.4) | 8.05E-01 | 9.09E-01 |
|  | HF | 94 | 1.12 (0.87-1.44) | 3.72E-01 | 6.00E-01 |
|  | AF | 93 | 1.05 (0.83-1.34) | 6.80E-01 | 8.29E-01 |
|  | Stroke | 93 | 0.94 (0.7-1.25) | 6.53E-01 | 8.29E-01 |
| Snoring | CAD | 242 | 1.04 (0.98-1.1) | 1.57E-01 | 4.36E-01 |
|  | MI | 237 | 1 (0.94-1.07) | 9.30E-01 | 9.69E-01 |
|  | HF | 241 | 1.02 (0.98-1.07) | 3.06E-01 | 5.46E-01 |
|  | AF | 235 | 1.01 (0.96-1.06) | 7.47E-01 | 8.69E-01 |
|  | Stroke | 245 | 1.01 (0.95-1.06) | 8.18E-01 | 9.09E-01 |
| Apnea–hypopnea index | CAD | 61 | 1.02 (0.99-1.05) | 2.40E-01 | 5.31E-01 |
|  | MI | 60 | 1.01 (0.98-1.04) | 5.68E-01 | 7.68E-01 |
|  | HF | 58 | 1.01 (0.99-1.04) | 3.38E-01 | 5.65E-01 |
|  | AF | 60 | 0.99 (0.97-1.01) | 3.86E-01 | 6.03E-01 |
|  | Stroke | 59 | 1.03 (0.99-1.06) | 1.08E-01 | 3.44E-01 |

CAD, coronary artery disease; MI, myocardial infarction; HF, heart failure; AF, atrial fibrillation. The OR represented the effect of genetically predicted per unit increase in each sleep trait.

## Table S13. Verification results of potentially weak instrumental bias in causal effects of sleep traits on MDD.

| Exposure | Mediator | Number of SNPs | Radial IVW | | |
| --- | --- | --- | --- | --- | --- |
|  |  |  | OR (95% CI) | *P*-value | *q*-value |
| Insomnia | MDD | 273 | 2.07 (1.76-2.43) | 8.94E-19 | 8.94E-18 |
| Sleep duration | MDD | 320 | 0.85 (0.76-0.93) | 9.73E-04 | 2.43E-03 |
| Short sleep | MDD | 234 | 1.83 (1.4-2.39) | 1.02E-05 | 5.10E-05 |
| Long sleep | MDD | 147 | 1.74 (1.1-2.77) | 1.80E-02 | 3.00E-02 |
| Daytime napping | MDD | 78 | 1.42 (1.06-1.89) | 1.70E-02 | 3.00E-02 |
| Daytime sleepiness | MDD | 241 | 1.67 (1.28-2.16) | 1.30E-04 | 4.33E-04 |
| Chronotype | MDD | 116 | 0.91 (0.83-0.99) | 3.32E-02 | 4.15E-02 |
| Morning person | MDD | 94 | 0.74 (0.57-0.96) | 2.28E-02 | 3.26E-02 |
| Snoring | MDD | 242 | 0.97 (0.92-1.02) | 2.57E-01 | 2.86E-01 |
| Apnea–hypopnea index | MDD | 61 | 1.01 (0.98-1.04) | 4.14E-01 | 4.14E-01 |

MDD, major depressive disorder. The OR represented the effect of genetically predicted per unit increase in each sleep trait.

## Table S14. Verification results of potentially weak instrumental bias in causal effects of MDD on CVDs.

| Mediator | Outcome | Number of SNPs | Radial IVW | | |
| --- | --- | --- | --- | --- | --- |
|  |  |  | OR (95% CI) | *P*-value | *q*-value |
| MDD | CAD | 181 | 1.06 (1.02-1.1) | 3.89E-03 | 6.57E-03 |
| MDD | MI | 178 | 1.06 (1.02-1.11) | 3.94E-03 | 6.57E-03 |
| MDD | HF | 176 | 1.07 (1.03-1.1) | 1.12E-04 | 5.60E-04 |
| MDD | AF | 182 | 1.01 (0.98-1.04) | 4.75E-01 | 4.75E-01 |
| MDD | Stroke | 184 | 1.05 (1.01-1.09) | 1.20E-02 | 1.50E-02 |

MDD, major depressive disorder; CAD, coronary artery disease; MI, myocardial infarction; HF, heart failure; AF, atrial fibrillation. The OR represented the effect of genetically predicted per log odds increase in MDD.

## Table S15. Replicate MR analyses results of the effect of sleep traits on CVDs screened by strict threshold criteria.

| Exposure* | Outcome | No. SNPs | MR-Egger intercept test *P* | Heterogeneity test | | IVW method‡ | OR (95% CI) | *P*-value | *q*-value |
| --- | --- | --- | --- | --- | --- | --- | --- | --- | --- |
|  |  |  |  | I^2^, %† | Q_*P* |  |  |  |  |
| Insomnia | CAD | 26 | 8.09E-01 | 29.30 | 8.19E-02 | fixed-effect IVW | 1.71 (1.14-2.56) | 1.02E-02 | 1.02E-01 |
|  | MI | 26 | 5.94E-01 | 34.21 | 4.63E-02 | random-effect IVW | 1.68 (0.97-2.92) | 6.57E-02 | 2.34E-01 |
|  | HF | 26 | 1.94E-01 | 32.30 | 5.86E-02 | fixed-effect IVW | 1.4 (1.01-1.95) | 4.63E-02 | 2.34E-01 |
|  | AF | 27 | 6.78E-02 | 12.60 | 2.78E-01 | fixed-effect IVW | 1.02 (0.75-1.39) | 8.82E-01 | 9.54E-01 |
|  | Stroke | 26 | 8.58E-01 | 46.51 | 5.27E-03 | random-effect IVW | 0.9 (0.53-1.53) | 6.99E-01 | 9.54E-01 |
| Sleep duration | CAD | 46 | 9.78E-01 | 29.37 | 3.45E-02 | random-effect IVW | 0.74 (0.59-0.92) | 8.13E-03 | 1.02E-01 |
|  | MI | 46 | 3.40E-01 | 19.31 | 1.30E-01 | fixed-effect IVW | 0.79 (0.64-0.98) | 3.01E-02 | 2.26E-01 |
|  | HF | 45 | 4.01E-01 | 0.00 | 6.42E-01 | fixed-effect IVW | 0.86 (0.74-1.01) | 6.61E-02 | 2.34E-01 |
|  | AF | 46 | 8.41E-01 | 48.14 | 1.84E-04 | random-effect IVW | 0.99 (0.81-1.21) | 9.54E-01 | 9.54E-01 |
|  | Stroke | 46 | 7.98E-01 | 0.00 | 5.78E-01 | fixed-effect IVW | 0.92 (0.76-1.11) | 3.73E-01 | 6.99E-01 |
| Short sleep | CAD | 15 | 2.69E-01 | 0.00 | 5.72E-01 | fixed-effect IVW | 3.55 (1.52-8.3) | 3.42E-03 | 1.02E-01 |
|  | MI | 15 | 8.31E-01 | 0.00 | 7.12E-01 | fixed-effect IVW | 2.59 (1.01-6.62) | 4.67E-02 | 2.34E-01 |
|  | HF | 15 | 7.55E-01 | 0.00 | 6.58E-01 | fixed-effect IVW | 1.12 (0.55-2.26) | 7.56E-01 | 9.54E-01 |
|  | AF | 15 | 3.89E-01 | 20.56 | 2.25E-01 | fixed-effect IVW | 1.15 (0.6-2.19) | 6.74E-01 | 9.54E-01 |
|  | Stroke | 15 | 2.76E-01 | 0.00 | 9.10E-01 | fixed-effect IVW | 1.3 (0.57-2.96) | 5.38E-01 | 8.97E-01 |
| Long sleep | CAD | 6 | 3.03E-01 | 0.00 | 4.83E-01 | fixed-effect IVW | 0.38 (0.06-2.61) | 3.26E-01 | 6.52E-01 |
|  | MI | 6 | 1.79E-01 | 25.85 | 2.40E-01 | fixed-effect IVW | 0.9 (0.11-7.44) | 9.22E-01 | 9.54E-01 |
|  | HF | 4 | 7.21E-01 | 0.00 | 5.36E-01 | fixed-effect IVW | 0.71 (0.13-3.94) | 6.91E-01 | 9.54E-01 |
|  | AF | 4 | 1.77E-01 | 36.97 | 1.90E-01 | fixed-effect IVW | 4.3 (0.89-20.91) | 7.03E-02 | 2.34E-01 |
|  | Stroke | 6 | 4.62E-01 | 0.00 | 9.07E-01 | fixed-effect IVW | 0.36 (0.05-2.65) | 3.13E-01 | 6.52E-01 |
| Daytime sleepiness | CAD | 22 | 1.59E-01 | 8.15 | 3.51E-01 | fixed-effect IVW | 1.53 (0.83-2.8) | 1.73E-01 | 4.72E-01 |
|  | MI | 23 | 2.53E-01 | 24.76 | 1.38E-01 | fixed-effect IVW | 1.08 (0.56-2.08) | 8.24E-01 | 9.54E-01 |
|  | HF | 22 | 5.15E-01 | 42.07 | 2.05E-02 | random-effect IVW | 1.6 (0.83-3.06) | 1.60E-01 | 4.72E-01 |
|  | AF | 21 | 9.11E-01 | 39.86 | 3.16E-02 | random-effect IVW | 1.44 (0.79-2.61) | 2.37E-01 | 5.47E-01 |
|  | Stroke | 23 | 1.34E-01 | 17.78 | 2.21E-01 | fixed-effect IVW | 0.96 (0.54-1.7) | 8.81E-01 | 9.54E-01 |
| Soring | CAD | 25 | 5.59E-01 | 20.32 | 1.81E-01 | fixed-effect IVW | 1.05 (0.93-1.18) | 4.73E-01 | 8.35E-01 |
|  | MI | 25 | 7.69E-01 | 31.21 | 7.00E-02 | fixed-effect IVW | 1.02 (0.89-1.16) | 7.93E-01 | 9.54E-01 |
|  | HF | 25 | 2.48E-01 | 39.34 | 2.38E-02 | random-effect IVW | 1.01 (0.89-1.15) | 8.31E-01 | 9.54E-01 |
|  | AF | 23 | 6.12E-01 | 23.76 | 1.49E-01 | fixed-effect IVW | 0.94 (0.86-1.04) | 2.17E-01 | 5.43E-01 |
|  | Stroke | 26 | 6.20E-01 | 12.54 | 2.82E-01 | fixed-effect IVW | 1.01 (0.9-1.13) | 8.92E-01 | 9.54E-01 |

CAD, coronary artery disease; MI, myocardial infarction; HF, heart failure; AF, atrial fibrillation. The odds ratio (OR) represented the effect of genetically predicted per unit increase in each sleep trait.

*Since daytime napping, chronotype and morning person have used strict threshold standards in main analysis, they will not be shown here.

†The formula for I^2^ is (Q-DF) /Q;

‡When there was significant heterogeneity (I^2^ > 25% and *P* < 0.05), the random-effect IVW model was used, otherwise the fixed-effect IVW model was used.

## Table S16. Replicate MR analyses results of the effect of sleep traits on MDD screened by strict threshold criteria.

| Exposure* | Mediator | No. SNPs | MR-Egger intercept test | Heterogeneity test | | IVW method‡ | OR (95% CI) | *P*-value | *q*-value |
| --- | --- | --- | --- | --- | --- | --- | --- | --- | --- |
|  |  |  |  | I^2^, % † | Q_*P* |  |  |  |  |
| Insomnia | MDD | 26 | 2.83E-01 | 50.53 | 1.83E-03 | random-effect IVW | 2.22 (1.38-3.58) | 1.02E-03 | 6.11E-03 |
| Sleep duration | MDD | 47 | 6.59E-01 | 59.13 | 1.68E-07 | random-effect IVW | 0.94 (0.73-1.21) | 6.34E-01 | 7.60E-01 |
| Short sleep | MDD | 15 | 6.36E-01 | 67.12 | 1.00E-04 | random-effect IVW | 1.75 (0.5-6.18) | 3.82E-01 | 7.60E-01 |
| Long sleep | MDD | 7 | 7.09E-01 | 30.18 | 1.98E-01 | fixed-effect IVW | 1.21 (0.23-6.27) | 8.19E-01 | 8.19E-01 |
| Daytime sleepiness | MDD | 23 | 6.66E-01 | 22.41 | 1.64E-01 | fixed-effect IVW | 1.79 (1.09-2.95) | 2.16E-02 | 6.49E-02 |
| Soring | MDD | 25 | 8.80E-02 | 54.12 | 7.12E-04 | random-effect IVW | 0.96 (0.83-1.12) | 6.33E-01 | 7.60E-01 |

MDD, major depressive disorder.The odds ratio (OR) represented the effect of genetically predicted per unit increase in each sleep trait.

*Since daytime napping, chronotype and morning person have used strict threshold standards in main analysis, they will not be shown here.

†The formula for I^2^ is (Q-DF) /Q;

‡When there was significant heterogeneity (I^2^ > 25% and *P* < 0.05), the random-effect IVW model was used, otherwise the fixed-effect IVW model was used.

## Table S17. Replicate MR analyses results of the effect of MDD on CVDs screened by strict threshold criteria.

| Mediator | Outcome | No. SNPs | MR-Egger intercept test‡ | Heterogeneity test | | IVW method† | OR (95% CI) | *P*-value | *q*-value |
| --- | --- | --- | --- | --- | --- | --- | --- | --- | --- |
|  |  |  |  | I^2^, %* | Q_*P* |  |  |  |  |
| MDD | CAD | 2 | - | 0 | 8.47E-01 | fixed-effect IVW | 1.21 (0.91-1.61) | 1.95E-01 | 2.44E-01 |
| MDD | MI | 2 | - | 0 | 8.52E-01 | fixed-effect IVW | 1.4 (1.02-1.92) | 3.65E-02 | 1.15E-01 |
| MDD | HF | 2 | - | 0.07 | 2.99E-01 | fixed-effect IVW | 1.27 (1-1.61) | 4.60E-02 | 1.15E-01 |
| MDD | AF | 2 | - | 0 | 3.34E-01 | fixed-effect IVW | 1.19 (0.95-1.49) | 1.21E-01 | 2.02E-01 |
| MDD | Stroke | 2 | - | 0 | 6.98E-01 | fixed-effect IVW | 1.04 (0.79-1.38) | 7.62E-01 | 7.62E-01 |

MDD, major depressive disorder; CAD, coronary artery disease; MI, myocardial infarction; HF, heart failure; AF, atrial fibrillation. The odds ratio (OR) represented the effect of genetically predicted per log odds increase in MDD.

*The formula for I^2^ is (Q-DF) /Q;

†When there was significant heterogeneity (I^2^ > 25% and *P* < 0.05), the random-effect IVW model was used, otherwise the fixed-effect IVW model was used.

‡Since SNP < 3, pleiotropy cannot be estimated.

## Table S18. The multiple multivariable MR analyses results of effects of MDD on each CVDs after adjusting for each sleep trait.

| Mediator | Adjustment factors | Outcome | No. SNPs | Multivariable IVW | | Multivariable MR Egger | | QHET method† |
| --- | --- | --- | --- | --- | --- | --- | --- | --- |
|  |  |  |  | OR (95% CI) | *P*-value | OR (95% CI) | *P*-value | OR |
| MDD | Insomnia | CAD | 451 | 1.05 (1.01-1.09) | 1.24E-02 | 1.05 (1.01-1.09) | 1.20E-02 | 1.04 |
|  |  | MI | 446 | 1.06 (1.02-1.10) | 7.21E-03 | 1.06 (1.02-1.10) | 7.00E-03 | 1.05 |
|  |  | HF | 443 | 1.06 (1.02-1.09) | 1.30E-03 | 1.06 (1.02-1.09) | 1.00E-03 | 1.05 |
|  |  | AF | 459 | 1.01 (0.98-1.04) | 5.85E-01 | 1.01 (0.98-1.04) | 6.56E-01 | 0.96 |
|  |  | AS | 455 | 1.04 (1.00-1.08) | 3.56E-02 | 1.04 (1.00-1.08) | 3.40E-02 | 1.05 |
| MDD | Sleep duration | CAD | 498 | 1.06 (1.02-1.10) | 1.34E-03 | 1.06 (1.03-1.10) | 1.00E-03 | 1.05 |
|  |  | MI | 496 | 1.07 (1.03-1.11) | 5.74E-04 | 1.07 (1.03-1.11) | 0.00E+00 | 1.04 |
|  |  | HF | 490 | 1.07 (1.04-1.10) | 2.30E-05 | 1.07 (1.04-1.10) | 0.00E+00 | 1.06 |
|  |  | AF | 497 | 1.02 (0.99-1.05) | 1.94E-01 | 1.02 (0.99-1.05) | 2.08E-01 | 0.97 |
|  |  | AS | 499 | 1.06 (1.02-1.09) | 2.23E-03 | 1.06 (1.02-1.10) | 1.00E-03 | 1.07 |
| MDD | Short sleep | CAD | 409 | 1.07 (1.03-1.11) | 5.79E-04 | 1.06 (1.02-1.10) | 1.00E-03 | 1.06 |
|  |  | MI | 404 | 1.08 (1.04-1.12) | 2.44E-04 | 1.08 (1.03-1.12) | 0.00E+00 | 1.06 |
|  |  | HF | 403 | 1.07 (1.04-1.10) | 2.32E-05 | 1.07 (1.04-1.10) | 0.00E+00 | 1.06 |
|  |  | AF | 411 | 1.01 (0.98-1.04) | 4.13E-01 | 1.01 (0.98-1.04) | 5.24E-01 | 0.97 |
|  |  | AS | 413 | 1.05 (1.02-1.09) | 3.28E-03 | 1.05 (1.02-1.09) | 4.00E-03 | 1.07 |
| MDD | Long sleep | CAD | 323 | 1.06 (1.02-1.10) | 3.41E-03 | 1.06 (1.02-1.10) | 5.00E-03 | 1.05 |
|  |  | MI | 318 | 1.06 (1.02-1.11) | 4.13E-03 | 1.06 (1.02-1.10) | 5.00E-03 | 1.03 |
|  |  | HF | 312 | 1.06 (1.02-1.09) | 8.28E-04 | 1.06 (1.02-1.09) | 1.00E-03 | 1.06 |
|  |  | AF | 325 | 1.00 (0.98-1.03) | 7.51E-01 | 1.01 (0.98-1.03) | 7.39E-01 | 0.97 |
|  |  | AS | 325 | 1.04 (1.01-1.08) | 1.79E-02 | 1.04 (1.01-1.08) | 2.40E-02 | 1.06 |
| MDD | Daytime napping | CAD | 261 | 1.05 (1.01-1.09) | 1.06E-02 | 1.05 (1.01-1.09) | 1.20E-02 | 1.05 |
|  |  | MI | 258 | 1.06 (1.01-1.11) | 1.06E-02 | 1.06 (1.01-1.11) | 1.50E-02 | 1.04 |
|  |  | HF | 256 | 1.06 (1.03-1.10) | 2.64E-04 | 1.06 (1.03-1.10) | 0.00E+00 | 1.06 |
|  |  | AF | 260 | 1.01 (0.98-1.04) | 4.90E-01 | 1.01 (0.98-1.04) | 4.75E-01 | 0.97 |
|  |  | AS | 264 | 1.05 (1.01-1.09) | 1.78E-02 | 1.05 (1.01-1.09) | 1.40E-02 | 1.07 |
| MDD | Daytime sleepiness | CAD | 415 | 1.05 (1.01-1.09) | 5.66E-03 | 1.05 (1.02-1.09) | 4.00E-03 | 1.05 |
|  |  | MI | 412 | 1.06 (1.01-1.10) | 7.93E-03 | 1.06 (1.02-1.10) | 6.00E-03 | 1.04 |
|  |  | HF | 405 | 1.07 (1.03-1.10) | 1.16E-04 | 1.07 (1.03-1.10) | 0.00E+00 | 1.06 |
|  |  | AF | 420 | 1.02 (0.99-1.05) | 1.70E-01 | 1.02 (0.99-1.05) | 1.16E-01 | 0.97 |
|  |  | AS | 419 | 1.05 (1.01-1.08) | 7.24E-03 | 1.05 (1.01-1.09) | 7.00E-03 | 1.07 |
| MDD | Chronotype | CAD | 296 | 1.06 (1.02-1.10) | 5.20E-03 | 1.06 (1.02-1.10) | 5.00E-03 | 1.05 |
|  |  | MI | 293 | 1.06 (1.02-1.11) | 5.40E-03 | 1.06 (1.02-1.11) | 5.00E-03 | 1.04 |
|  |  | HF | 292 | 1.07 (1.03-1.10) | 1.12E-04 | 1.07 (1.03-1.10) | 0.00E+00 | 1.06 |
|  |  | AF | 298 | 1.01 (0.98-1.04) | 4.97E-01 | 1.01 (0.98-1.04) | 4.97E-01 | 0.97 |
|  |  | AS | 299 | 1.04 (1.01-1.08) | 1.94E-02 | 1.04 (1.01-1.08) | 1.80E-02 | 1.06 |
| MDD | Morning person | CAD | 274 | 1.06 (1.02-1.10) | 4.92E-03 | 1.06 (1.02-1.10) | 5.00E-03 | 1.05 |
|  |  | MI | 271 | 1.06 (1.02-1.11) | 3.72E-03 | 1.06 (1.02-1.11) | 4.00E-03 | 1.04 |
|  |  | HF | 270 | 1.07 (1.03-1.10) | 1.19E-04 | 1.07 (1.03-1.10) | 0.00E+00 | 1.06 |
|  |  | AF | 275 | 1.01 (0.99-1.04) | 3.56E-01 | 1.01 (0.98-1.04) | 3.61E-01 | 0.97 |
|  |  | AS | 277 | 1.05 (1.01-1.09) | 8.15E-03 | 1.05 (1.01-1.09) | 8.00E-03 | 1.07 |
| MDD | Snoring | CAD | 423 | 1.05 (1.01-1.09) | 7.98E-03 | 1.05 (1.01-1.09) | 8.00E-03 | 1.05 |
|  |  | MI | 415 | 1.06 (1.01-1.10) | 9.58E-03 | 1.05 (1.01-1.10) | 1.60E-02 | 1.04 |
|  |  | HF | 417 | 1.06 (1.03-1.10) | 2.84E-04 | 1.06 (1.03-1.09) | 0.00E+00 | 1.07 |
|  |  | AF | 417 | 1.01 (0.98-1.04) | 4.73E-01 | 1.01 (0.98-1.04) | 4.74E-01 | 0.97 |
|  |  | AS | 429 | 1.04 (1.01-1.08) | 2.02E-02 | 1.04 (1.01-1.08) | 1.80E-02 | 1.06 |
| MDD | Apnea–hypopnea index | CAD | 242 | 1.06 (1.02-1.10) | 5.31E-03 | 1.06 (1.02-1.10) | 5.00E-03 | 1.05 |
|  |  | MI | 238 | 1.06 (1.02-1.11) | 4.41E-03 | 1.06 (1.02-1.11) | 4.00E-03 | 1.04 |
|  |  | HF | 234 | 1.07 (1.03-1.10) | 1.99E-04 | 1.07 (1.03-1.10) | 0.00E+00 | 1.06 |
|  |  | AF* | 242 | 1.01 (0.99-1.04) | 3.39E-01 | 1.01 (0.99-1.04) | 3.71E-01 | 0.98 |
|  |  | AS | 243 | 1.05 (1.01-1.08) | 1.92E-02 | 1.04 (1.01-1.08) | 1.90E-02 | 1.07 |

MDD, major depressive disorder; CAD, coronary artery disease; MI, myocardial infarction; HF, heart failure; AF, atrial fibrillation. The odds ratio (OR) represented the effect of genetically predicted per log odds increase in MDD.

*Since the MR-egger intercept test showed the existence of pleiotropy, the multivariable MR-egger result was the main results. There was no heterogeneity and pleiotropy in other, so the multivariable IVW result was the main results.

†Since the QHET model is currently under development and the 95% confidence interval is not accurate, only the point estimation results are displayed.


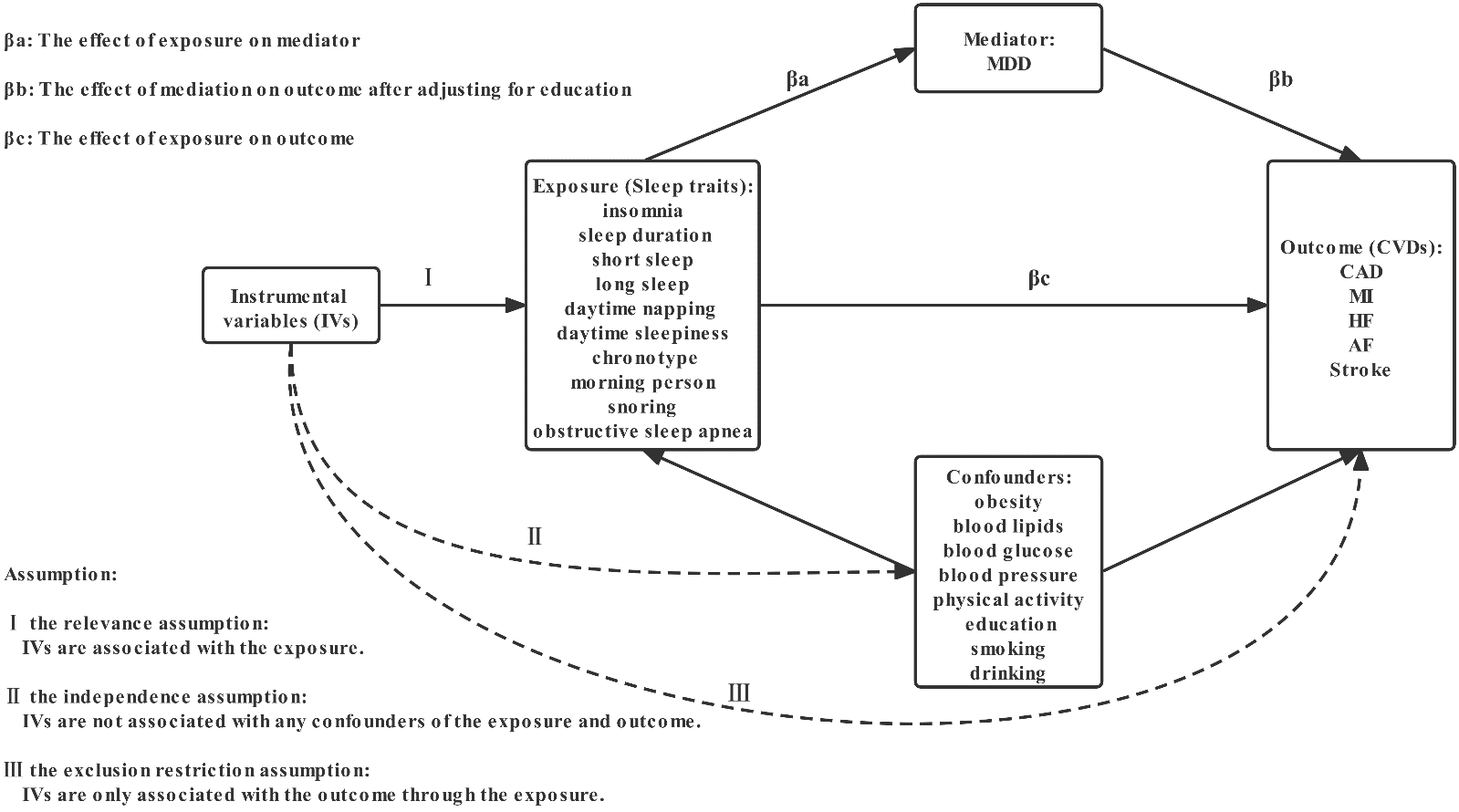


## Figure S1. The assumption of MR model.

MDD, major depressive disorder; MR, Mendelian randomization; CAD, coronary artery disease; MI, myocardial infarction; HF, heart failure; AF, atrial fibrillation.


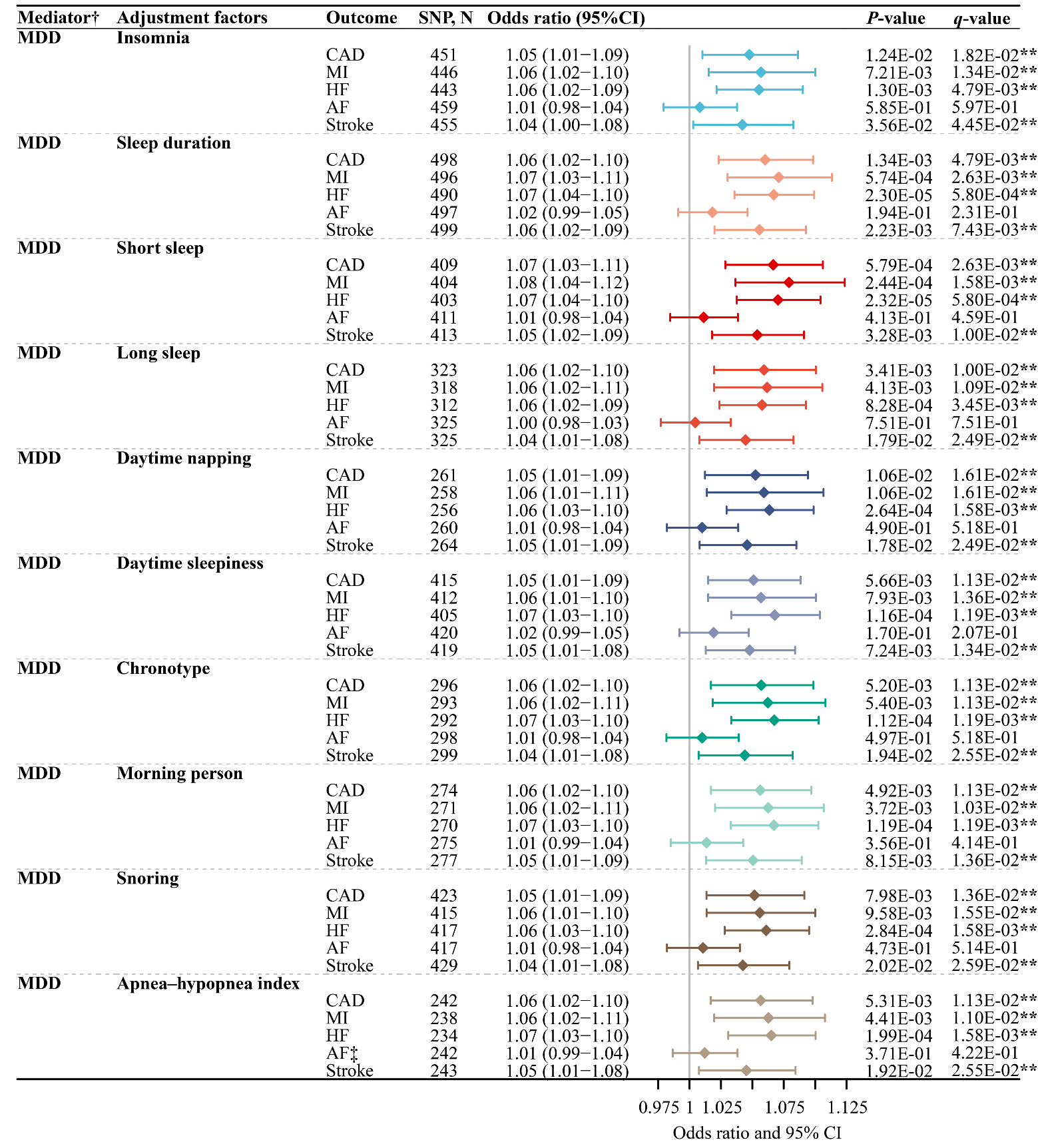


**Figure** S2**. The main MR analysis results of the causal effects of MDD on CVDs after adjusting for each sleep trait.**

The OR represented the effect of genetically predicted per unit (log odds) increase in MDD.

(**): Indicates significant evidence (*q*-value <0.05) after correction for multiple testing estimated by the FDR method.

(*): Indicates suggestive evidence (uncorrected *P*-value <0.05 and *q*-value ≥0.05).

(†): Because there was significant pleiotropy, the multivariable MR-Egger model was used, while for others, because there was no significant pleiotropy and heterogeneity, the multivariable IVW model was used.

MDD, major depressive disorder; CAD, coronary artery disease; MI, myocardial infarction; HF, heart failure; AF, atrial fibrillation

## References

1. Lane JM, Jones SE, Dashti HS, et al. Biological and clinical insights from genetics of insomnia symptoms. *Nat Genet* 2019;51:387-93.

2. Dashti HS, Jones SE, Wood AR, et al. Genome-wide association study identifies genetic loci for self-reported habitual sleep duration supported by accelerometer-derived estimates. *Nat Commun* 2019;10:1100.

3. Dashti HS, Daghlas I, Lane JM, et al. Genetic determinants of daytime napping and effects on cardiometabolic health. *Nat Commun* 2021;12:900.

4. Wang H, Lane JM, Jones SE, et al. Genome-wide association analysis of self-reported daytime sleepiness identifies 42 loci that suggest biological subtypes. *Nat Commun* 2019;10:3503.

5. Jones SE, Lane JM, Wood AR, et al. Genome-wide association analyses of chronotype in 697,828 individuals provides insights into circadian rhythms. *Nat Commun* 2019;10:343.

6. Jansen PR, Watanabe K, Stringer S, et al. Genome-wide analysis of insomnia in 1,331,010 individuals identifies new risk loci and functional pathways. *Nat Genet* 2019;51:394-403.

7. Chen H, Cade BE, Gleason KJ, et al. Multiethnic Meta-Analysis Identifies RAI1 as a Possible Obstructive Sleep Apnea-related Quantitative Trait Locus in Men. *Am J Respir Cell Mol Biol* 2018;58:391-401.

8. Pierce BL, Ahsan H, Vanderweele TJ. Power and instrument strength requirements for Mendelian randomization studies using multiple genetic variants. *Int J Epidemiol* 2011;40:740-52.

9. Rosoff DB, Smith GD, Lohoff FW. Prescription Opioid Use and Risk for Major Depressive Disorder and Anxiety and Stress-Related Disorders: A Multivariable Mendelian Randomization Analysis. *JAMA Psychiatry* 2021;78:151-60.

10. Jones DP, Wootton RE, Gill D, et al. Mental Health as a Mediator of the Association Between Educational Inequality and Cardiovascular Disease: A Mendelian Randomization Study. *J Am Heart Assoc* 2021;10:e019340.

11. Kamat MA, Blackshaw JA, Young R, et al. PhenoScanner V2: an expanded tool for searching human genotype-phenotype associations. *Bioinformatics* 2019;35:4851-53.

12. Machiela MJ, Chanock SJ. LDlink: a web-based application for exploring population-specific haplotype structure and linking correlated alleles of possible functional variants. *Bioinformatics* 2015;31:3555-7.

13. Leong A, Cole JB, Brenner LN, et al. Cardiometabolic risk factors for COVID-19 susceptibility and severity: A Mendelian randomization analysis. *PLoS Med* 2021;18:e1003553.

14. Verbanck M, Chen CY, Neale B, et al. Detection of widespread horizontal pleiotropy in causal relationships inferred from Mendelian randomization between complex traits and diseases. *Nat Genet* 2018;50:693-98.

15. Brion MJ, Shakhbazov K, Visscher PM. Calculating statistical power in Mendelian randomization studies. *Int J Epidemiol* 2013;42:1497-501.

16. Xue H, Shen X, Pan W. Constrained maximum likelihood-based Mendelian randomization robust to both correlated and uncorrelated pleiotropic effects. *Am J Hum Genet* 2021;108:1251-69.

17. Bowden J, Davey Smith G, Burgess S. Mendelian randomization with invalid instruments: effect estimation and bias detection through Egger regression. *Int J Epidemiol* 2015;44:512-25.

18. Barry C, Liu J, Richmond R, et al. Exploiting collider bias to apply two-sample summary data Mendelian randomization methods to one-sample individual level data. *PLoS Genet* 2021;17:e1009703.

19. Bowden J, Davey Smith G, Haycock PC, et al. Consistent Estimation in Mendelian Randomization with Some Invalid Instruments Using a Weighted Median Estimator. *Genet Epidemiol* 2016;40:304-14.

20. Bowden J, Spiller W, Del Greco MF, et al. Improving the visualization, interpretation and analysis of two-sample summary data Mendelian randomization via the Radial plot and Radial regression. *Int J Epidemiol* 2018;47:1264-78.

21. Sanderson E. Multivariable Mendelian Randomization and Mediation. *Cold Spring Harb Perspect Med* 2021;11

22. Sobel ME. Effect analysis and causation in linear structural equation models. *Psychometrika* 1990;55:495-515.

23. Nevo D, Liao X, Spiegelman D. Estimation and Inference for the Mediation Proportion. *Int J Biostat* 2017;13
